# Functional identification of *BpMYB21* and *BpMYB61* transcription factors responding to MeJA and SA in birch triterpenoid synthesis

**DOI:** 10.1186/s12870-020-02521-1

**Published:** 2020-08-12

**Authors:** Jing Yin, Lu Sun, Ying Li, Jialei Xiao, Siyao Wang, Jie Yang, Ziyue Qu, Yaguang Zhan

**Affiliations:** 1 Key Laboratory of Saline-alkali Vegetation Ecology Restoration, Ministry of Education (Northeast Forestry University), Harbin, 150040 China; 2 College of Life Science, Northeast Forestry University, Harbin, 150040 China; 3 State Key Laboratory of Tree Genetics and Breeding, Northeast Forestry University, Harbin, 150040 China; 4 College of Life Science, Northeast Agricultere University, Harbin, 150010 China

**Keywords:** *Betula platyphylla* Suk., MYB transcription factors, Expression analysis, Triterpenoids, Functional annotation

## Abstract

**Background:**

Triterpenoids from birch (*Betula platyphylla* Suk.) exert antitumor and anti-HIV activities. Due to the complexity of plant secondary metabolic pathways, triterpene compounds in plants is not always determined by a single gene; they may be controlled by polygene quantitative traits. Secondary metabolism related to terpenoids involves tissue specificity and localisation of key biosynthetic enzymes. Terpene synthesis is influenced by light, hormones and other signals, as well as upstream transcription factor regulation.

**Results:**

Anchor Herein, we identified and characterised two birch MYB transcription factors (TFs) that regulate triterpenoid biosynthesis. BpMYB21 and BpMYB61 are R2R3 TFs that positively and negatively regulate responses to methyl-jasmonate (MeJA) and salicyclic acid (SA), respectively. Expression of BpMYB21 and BpMYB61 was elevated in leaves and stems more than roots during July/August in Harbin, China. BpMYB21 expression was increased by abscisic acid (ABA), MeJA, SA and gibberellins (GAs). BpMYB61 expression in leaves and BpMYB21 expression in stems was reduced by ABA, MeJA and SA, while GAs, ethylene, and injury increased BpMYB61 expression. BpMYB21 was localised in nuclei, while BpMYB61 was detected in cell membranes and nuclei. Promoters for both BpMYB21 (1302 bp) and BpMYB61 (850 bp) were active. BpMYB21 and BpMYB61 were ligated into pYES3, introduced into AnchorINVScl (yeast strain without exogenous genes), INVScl-pYES2-SSAnchorAnchor (transgenic yeast strain harbouring the SS gene from birch), and INVScl-pYES2-SE (transgenic yeast strain harbouring the SE gene from birch), and the squalene content was highest in AnchorINVScl-pYES-MYB21-SS (transgenic yeast strain harbouring SS and MYB21 genes) and INVScl-pYES3-MYB61 (transgenic yeast strain harbouring the MYB61 gene). In BpMYB21 transgenic birch key triterpenoid synthesis genes were up-regulated, and in BpMYB61 transgenic birch AnchorFPS (farnesyl pyrophosphate synthase) and SS (squalene synthase) were up-regulated, but HMGR (3-hydroxy-3-methylglutaryl coenzyme a reductase), BPWAnchor (lupeol synthase), SE (squalene epoxidase) and BPY (b-amyrin synthase) were down-regulated. Both BpMYB21 and BpMYB61 specifically activate SE and BPX (cycloartenol synthase synthesis) promoters.

**Conclusions:**

These findings support further functional characterisation of R2R3-MYB genes, and illuminate the regulatory role of *BpMYB21* and *BpMYB61* in the synthesis of birch triterpenoids.

## Background

Birch (*Betula platyphylla* Suk.) is an important economic tree species in China, and its leaves and bark contain many secondary metabolites including triterpenes of *Betula platyphylla* (TBP) possessing wide-ranging pharmacological effects [1]. Triterpenes from birch bark have been linked to resistance to HIV and inhibition of tumours (gliomas, melanoma, cervical cancer, breast tumour, leukaemia, myeloma), with low toxicity and high efficacy, making them promising new drug candidates [2–7].

It is very difficult to improve content of triterpenoid using traditional breeding methods due to the complexity of secondary metabolic pathways. In recent years, plant molecular biology and genetic engineering has facilitated new ways to manipulate plant secondary metabolites. However, triterpenoids produced by plants are often determined not by a single gene, but by quantitative traits controlled by polygenes, and secondary metabolism of terpenoids is tissue-specific and related to the location of key enzymes involved in biosynthesis. The synthesis of triterpenes is also regulated by light, hormones, and upstream transcription factors (TFs) [1, 8]. Therefore, isolation and identification of TFs related to triterpene synthesis and the regulation of triterpene metabolism is important for improving triterpenoid production via genetic manipulation.

MYB TFs are one of the largest TF families in plants, and they exert comprehensive biological functions including responses to abiotic stresses such as low temperature, drought, salt stress, pathogens and insect pests, mediate light, hormones and signal transduction, and play an important role in plant growth and development, and secondary metabolism regulation [9, 10]. For example, MYB TFs can form transcriptional complexes with basic helix-loop-helix (bHLH) TFs and regulate stem development and seed formation in *Arabidopsis thaliana* under the mediation of jasmonic acid (JA) [11]. In plant secondary metabolism, phosphorylation of MYB75 by MPK4 promotes the accumulation of anthocyanins in *A. thaliana* mediated by light [12]. In the biosynthesis of flavonoids, MYB TFs can regulate the expression of enzyme-coding genes related to flavonoid biosynthesis, thereby effectively regulating the synthesis of flavonoids [13]. MYB TFs have been characterised in *A. thaliana*, wheat, *Fraxinus velutina* Torr., *Solanum lycopersicum* (tomato) and other plants [14]. Recently, new reactive oxygen species (ROS)- and defence-related R2R3-MYB genes haven been identified in canola (*Brassica napus* L.), and members involved in anthocyanin biosynthesis in red kiwifruit (*Actinidia chinensis*) have been reported [14, 15], but R2R3-MYB genes associated with triterpenes in birch have not been reported.

Methyl jasmonate (MeJA) is an important plant endogenous hormone that participates in the biosynthesis and metabolism of plant flavonoids [16] and acts as an external signalling molecule to induce and activate the phenylpropane metabolic pathway [17]. Previous research in our laboratory showed that MeJA treatment significantly promotes the accumulation of triterpenes in the bark and leaves of birch, and in cultured birch cells [8]. The combined effects of high temperature and MeJA on triterpenoid synthesis in birch cells are stronger than the individual effects of MeJA or high temperature alone [18]. Salicylic acid (SA) not only plays an important role in plant stress, disease, and insect and microbial infection, but also in stimulating the plant defence system to produce phytoprotective hormones, and it is also an important signalling molecule that mediates exogenous inducers to promote the synthesis of secondary metabolites in plants [19, 20]. The effects of MeJA and SA on the synthesis of terpenoids can rapidly induce the expression of specific biosynthetic genes with high specificity [21, 22].

Triterpenoids are synthesised from 2,3-oxidosqualene, the precursor of various types of triterpenes. The key enzymes 3-hydroxy-3-methyl glutaryl coenzyme A reductase (HMGR), farnesyl pyrophosphonate synthase (FPS), squalene synthase (SS), and squalene epoxidase (SE) are essential in triterpene synthesis [23, 24]. Previous studies have shown that MeJA and SA can significantly induce the expression of key genes in the MVA and triterpenoid synthesis pathways in birch cells [8, 25]. In the present study, we identified and characterised two MYB TFs (*BpMYB21* and *BpMYB61*) that respond to MeJA and SA induction. The promoters of *SE* (squalene epoxidase*)*, *BPW* (lupeol synthase) and *BPX* (cycloartenol synthase) in birch contain MYB binding sites regulated by MYB TFs (to be published), therefore we speculated that these MYB TFs responding to MeJA and SA may play an important role in triterpenoid synthesis. Based on previous experimental results, we cloned *BpMYB21* and *BpMYB61* genes based on our transcriptome database of birch genes induced by MeJA and SA. The TFs were tested for their responses to hormones and injury. The novel *BpMYB21* gene could promote triterpenoid biosynthesis when expressed in birch and yeast. Our results lay a solid foundation for further characterisation of MYB TFs involved in the synthesis of birch triterpenes.

## Results

### Isolation and characterisation of *BpMYB21* and *BpMYB61*

Herein, we isolated *MYB21* and *MYB61* from our birch transcriptome database and investigated their possible functions in triterpene biosynthesis. The full-length *BpMYB21*((Genebank ID MF574045, NCBI) and *BpMYB61* (Genebank ID KT344120, NCBI) genes were isolated by RT-PCR. The *BpMYB21* cDNA sequence is 1117 bp and contains a 1014 bp open reading frame (ORF) encoding a 337 amino acid polypeptide (Additional file 1). The *BpMYB61* cDNA sequence is 1203 bp and contains a 1203 bp ORF encoding a protein of 400 amino acids (Additional file 2).

**Fig. 1:**
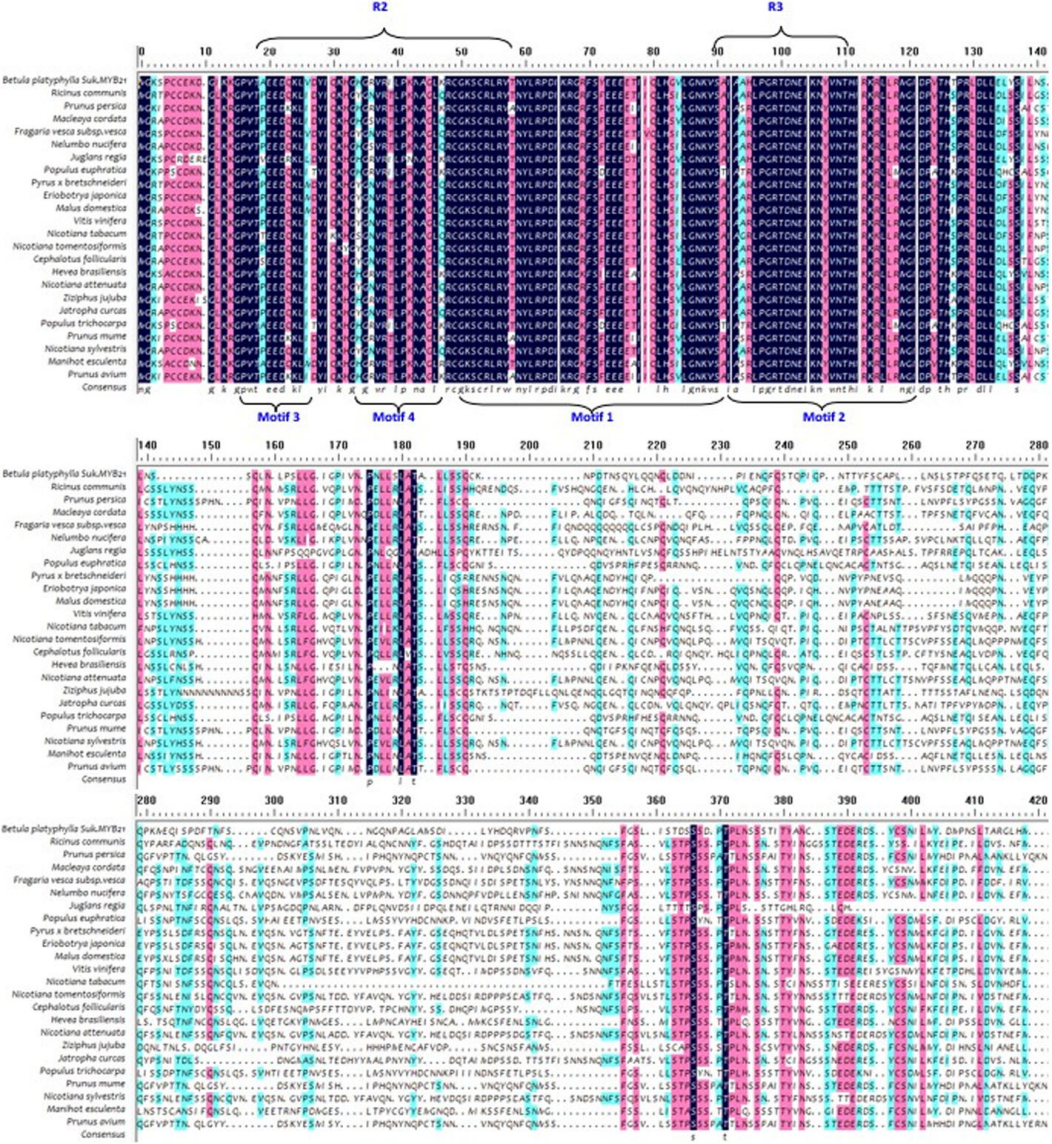
Multiple alignment of the amino acid sequence encoded by *BpMYB21* with those encoded by *MYB*s of other organisms obtained. Alignment was performed using the DNAMAN multiple alignment tool. Horizontal arrows represent R domains. Vertical arrows indicate the motif in the R2R3 domain that facilitates interaction with a MYB partner. The aligned sequences were derived from *Juglans regia* MYB30-Like (XP_018851905.1), *Vitis vinifera* MYB39 (XP_002281638.1), *Populus euphratica* MYB86 (XP_011044525.1), *Populus trichocarpa* (XP_006370357.1), *Nicotiana tabacum* MYB39 (OIT30152.1), *Nelumbo nucifera* MYB39 (XP_010277911.1) and *Fragaria vesca subsp. vesca* ODORANT1-like (XP_004293589.1)

**Fig. 2:**
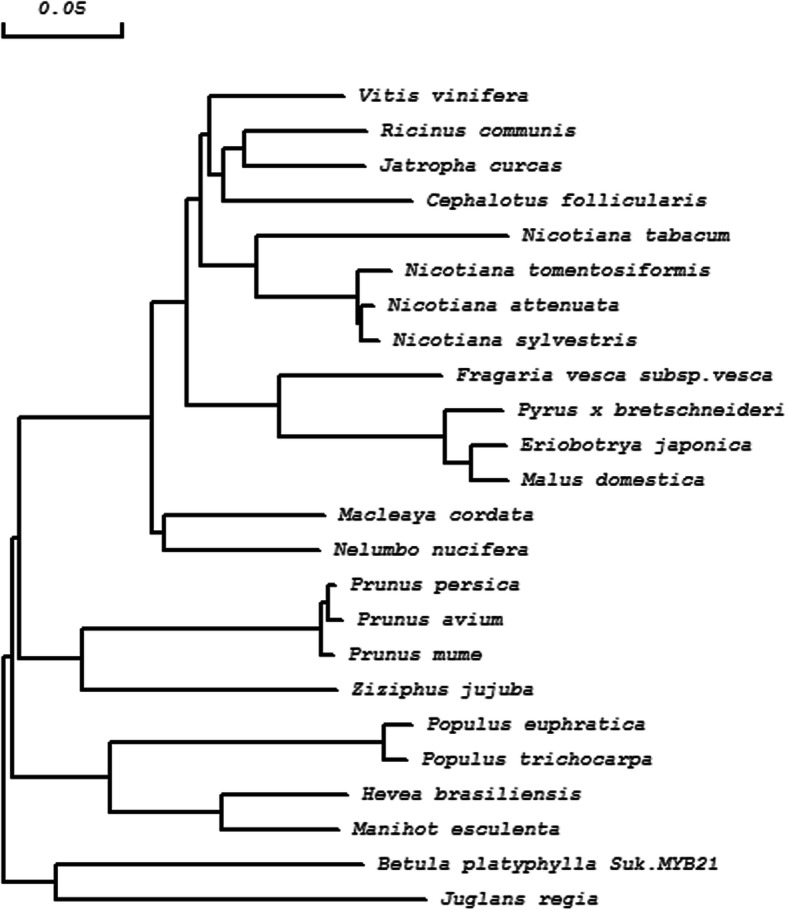
Neighbour-joining phylogenetic tree of *BpMYB21* from *Betula platyphylla* Suk. and other *MYBs* constructed using DNAMAN 8.0 software. The scale bar represents 0.05 amino acid substitutions per site

**Fig. 3:**
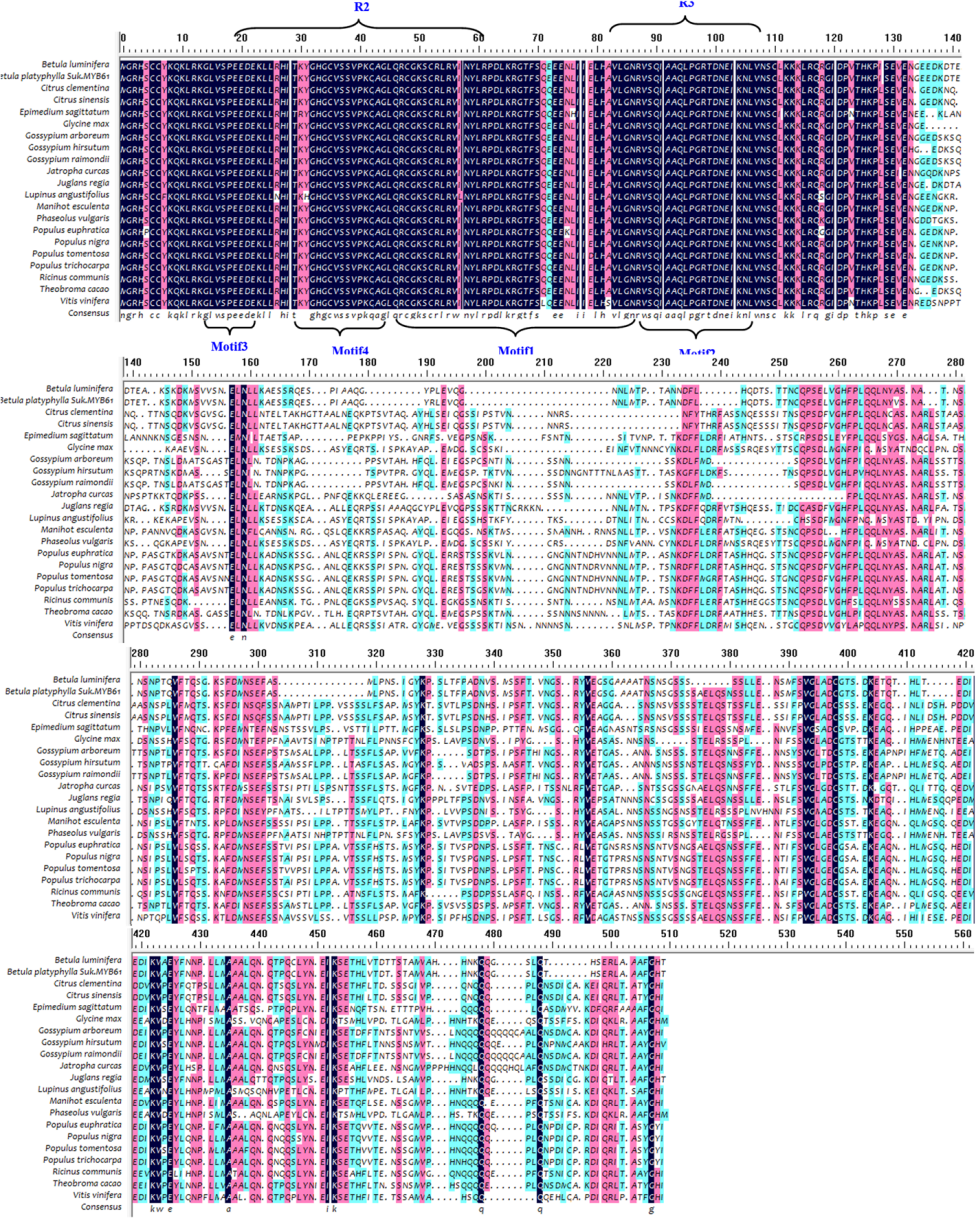
Multiple alignment of the amino acid sequence encoded by *BpMYB61* with those encoded by *MYB*s of other organisms. Alignment was performed using the DNAMAN multiple alignment tool. The aligned sequences were derived from *Betula luminifera* (ACJ38663.1), *Populus trichocarpa* (XP_002320929.1), *Juglans regia* (XP_018843144.1), *Populus euphratica* (XP_011040042.1), *Populus tomentosa* (AIA66961.1), *Populus nigra* (BAR45573.1), *Manihot esculenta* (OAY48816.1), *Theobroma cacao* (XP_007051325.1), *Vitis vinifera* (XP_002281027.1) and *Gossypium hirsutum* (XP_016695803.1)

R2R3-MYBs are characterised by R2 and R3 repeats [10], and our analysis confirmed the presence of the R domain and four motifs in the R2R3 domain that facilitate interaction with MYB partners (Figs. 1, 2 and 3).

Conserved domains of proteins encoded by *BpMYB21* and *BpMYB61* were predicted using the Conserved Domains function in BLAST, and the results indicated that BpMYB21 contains a Myb DNA-binding domain and two SANT-specific regions, spanning amino acid (aa) residues 14–61,14–63 and 16–61, respectively. The *BpMYB21* gene is a member of the R2R3-MYB family, and members of this family regulate flavonoid biosynthesis in various plant species. Physicochemical properties of the BpMYB21 protein were analysed using ProtParam on the ExPASy website, revealing a molecular mass of 38.04 kDA and an isoelectric point (pI) of 6.45. The instability coefficient is 49.59, implying it is an unstable protein (an instability coefficient < 40 indicates stability). The average hydrophilicity of the BpMYB21 protein is − 0.632, implying that it is hydrophilic. Hydrophilic amino acid sequence analysis using Protscale predicted a strongest hydrophobic value of 2.344 in position 164, and a strongest hydrophilic value of − 2.722 in position 34 (> 0.5 indicates hydrophobicity, < 0.5 indicates hydrophilicity, and a value between these indicates mixed properties). Prediction of transmembrane regions in BpMYB21 suggested that the N-terminus of the protein lies outside the membrane, and there are two transmembrane helices, spanning residues 138 to 161, (inner- to extracellular direction), and residues 146 to 164 (extra- to intracellular direction). Secondary structure prediction suggested that BpMYB21 consists of α-helices (26.41%), extended strands (11.28%), random coils (10.68%), and irregular turns (51.63%). The amino acid sequence of BpMYB21 shares ~ 64% sequence identity with MYBs from *Juglans regia*, *Populus euphratica*, and *Vitis vinifera*. Multiple sequence alignment of BpMYB21 with other MYB proteins is shown in Fig. 1, and a neighbour-joining phylogenetic tree constructed using DNAMAN 8.0 software indicates strongest relatedness with the homolog in *J. regia* (Fig. 2, Additional file 3).

**Fig. 4:**
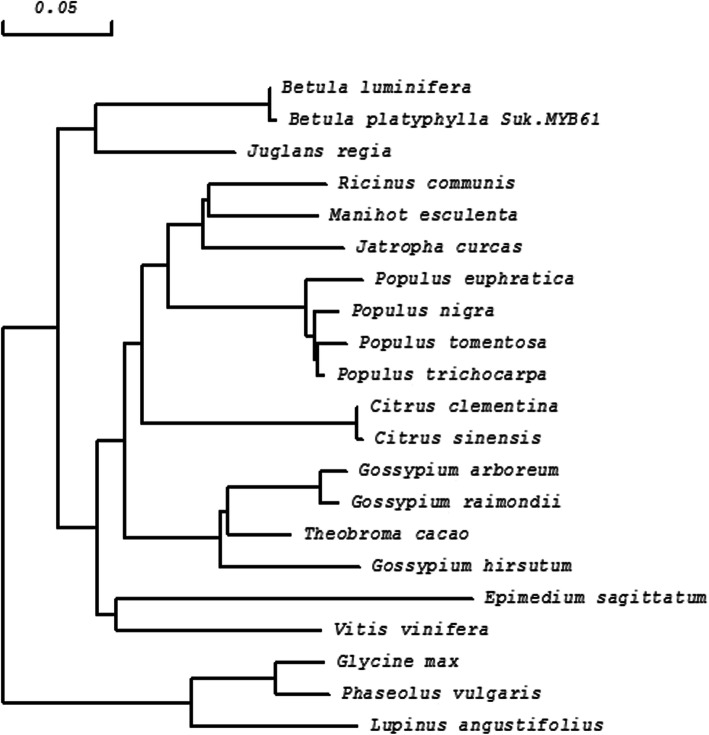
Neighbour-joining phylogenetic tree of *BpMYB61* from *Betula platyphylla* and other *MYBs* constructed using DNAMAN 8.0 software. The scale bar represents 0.05 amino acid substitutions per site

The *BpMYB61* gene is also a member of the R2R3-MYB family (Fig. 3), with R2 and R3 MYB DNA-binding residues spanning positions 14–61 and 67–112, respectively. Physicochemical properties of BpMYB61 were analysed using ProtParam on the ExPASy website, revealing a molecular mass of 44.77 kDA and a pI of 6.51. The instability coefficient is 51.11, implying an unstable protein. The average hydrophilicity is − 0.746, indicating a hydrophilic protein. Protscale predicted a strongest hydrophobic value of 2.189 for position 81, and a strongest hydrophilic value of − 2.967 for position 140. The BpMYB61 protein has no transmembrane helices. Secondary structural prediction using SOPMA software indicates that BpMYB61 consists of α-helices (44%), beta-turns (6.00%), extended strands (8.25%), and random coils (41.75%). The amino acid sequence of BpMYB61 shares 61–97% homology with MYBs from *Populus euphratica Oliv*, *Juglans regia*, *Populus trichocarpa*, and *Betula luminifera*. Multiple sequence alignment of BpMYB61and other MYB proteins was performed, and the results were used to construct a neighbour-joining phylogenetic tree with DNAMAN 8.0 software, which identified BpMYB61 from *B. luminifera* as the closest homolog (Figs. 3 and 4, Additional file 3).

### Isolation and analysis of *BpMYB21* and *BpMYB61* promoter sequences

The promoter sequences of *BpMYB21* and *BpMYB61* genes, 1302 and 850 bp, respectively, were amplified by the genome walking approach, and analysed using PlantCARE software. The results revealed the presence of TATA and CAAT boxes. Additionally, 5′-untranslated regions (5′UTRs) were identified at + 901 bp on the *BpMYB21* promoter, and + 688 bp on the *BpMYB61* promoter (Tables 1 and 2, Additional file 4).

**Table 1:** Putative cis-acting regulatory elements identified in the promoter sequence of *BpMYB21* using the Plant CARE database

Cis element	Position	Sequence	Function of site
5UTR Py-rich repeats	+ 901	TTTCTTCTCT	High efficiency transcriptional element
CAAT-box	− 42、 + 57、 + 87 etc.	CAAT、CAATT、CAAAT	Cis-regulatory elements in promoter and enhancer regions
TATA-box	+ 214、 + 255、 + 258、 + 297 etc.	TAATA、TATA、 ATTATA	Transcription initiation-30 core promoter element
ABRE	− 467、 + 469	CACGTG	Abscisic acid response element
ARE	+ 101	TGGTTT	Anaerobic-induced response element
Box-3	+ 570	atCATTTTCACt	Protein binding site
CGTCA-motif	+ 673	CGTCA	Methyl jasmonate response element
G-box	+ 469、-468、 + 671、	CACGTG	Light response element
GAG-motif	− 391、 + 746、-715	AGAGAGT	Partial light response element
GATA-motif	− 240	AAGGATAAGG	Partial light response element
HSE	+ 1216	AAAAAATTTC	Heat stress response element
I-box	+ 79、 + 704、-238	CTCTTATGCT	Partial light response element
MBS	+ 179、-462	CAACTG	MYB binding sites involving drought induced response
O2-site	− 670、 + 794、 + 749	GATGACATGA	Corn protein metabolic regulatory element
Skn-1-motif	+ 108、-858、-324	GTCAT	Endosperm expression cis-acting element
Sp1	+ 952、 + 1246	CC(G/A)CCC	Light response element
TC-rich repeats	+ 622	GTTTTCTTAC	Defense and stress response elements
TCA-element	− 960	CAGAAAAGGA	Salicylic acid induced response element
TCCC-motif	− 744	TCTCCCT	Partial Light response element
TGACG-motif	−673	TGACG	Methyl jasmonate induced response element
WUN-motif	+ 226	TCATTACGAA	Injury response element
circadian	+ 142	CAANNNNATC	Rhythm control element

**Table 2:** Putative cis-acting regulatory elements identified in the promoter sequence of *BpMYB61* using the Plant CARE database

Cis element	Position	Sequence	Function of site
5UTR Py-rich stretch	+ 680、 + 688、 + 684、 + 682、 + 686	TTTCTCTCTCTCTC	High efficiency transcriptional element
ARE	+ 282、-443	TGGTTT	Anaerobic-induced response element
Box I	− 126	TTTCAAA	Light response element
CAAT-box	+ 86、-101、-102、-125、 + 226 etc.	CAAT、CAATT、CCAAT	Cis-regulatory elements in promoter and enhancer
CAT-box	− 471	GCCACT	Meristem expression regulatory element
CCAAT-box	− 761	CAACGG	MYBHv1 binding site
G-box	+ 251、-270	GCCTTGTGTAG	Light response element
GA-motif	− 626	AAAGATGA	Partial light response element
GAG-motif	+ 397、-672	AGAGATG	Partial light response element
GARE-motif	+ 599	AAACAGA	Gibberellin response element
GCN4_motif	+ 34、-250、-94	CAAGCCA	Endosperm expression element
I-box	− 551	CTCTTATGCT	Partial light response element
MBS	+ 586	TAACTG	MYB binding sites involving drought induced response
O2-site	+ 87	GATGACATGG	Corn protein metabolic regulatory element
RY-element	+ 329	CATGCATG	Seed specific expression regulatory element
Skn-1_motif	−88	GTCAT	Endosperm expression element
Sp1	+ 135	CC(G/A)CCC	Light response element
TATA-box	− 78、-502、-339、 + 804、 + 182 etc.	TTTTA、TATA、TATTTAAA	Transcription initiation-30 core promoter element
TCA-element	− 395	CCATCTTTTT	Salicylic acid response element
TCCC-motif	+ 674、 + 818	TCTCCCT	Partial light response element
Unnamed__4	−22、 + 727、 + 675、 + 819 etc.	CTCC	
box E	− 306	ACCCATCAAG	
box S	+ 749	AGCCACC	
chs-Unit 1 m1	+192	ACCTAACCTCC	Partial light response element
circadian	−234	CAANNNNATC	Rhythm control element

In the *BpMYB21* promoter, three components related to hormone regulation were identified; a JA response element (CGTCA motif) at + 673 bp, two ABA response elements (ABRE motifs) at − 467 and + 469 bp, and an SA response element (TCA element) at − 960 bp. In addition, several regulatory elements related to light regulation were identified in the *BpMYB21* promoter, including GAG, SP1, I-box, GATA and G-box motifs, all related to the optical responses of cis-elements. Six endosperm expression elements (Skn-1 and GCN4 motifs) were found at + 108, − 858 and − 324 bp, + 34 bp, − 250 bp and − 94 bp. Additionally, the *BpMYB21* promoter contains MBS and TC-rich repeat elements at + 179, − 462 and + 622 bp, respectively. MBS can be combined with MYB TFs related to drought induction, and TC-rich repeats are cis-acting elements that operate in response to adverse environmental conditions and in defence mechanisms (Additional file 4).

In the *BpMYB61* promoter, two anaerobe-induced regulatory elements (AREs) were found at + 282 and − 443 bp, along with four light response elements (BoxI, G-box, and Sp1) located at + 126, + 135, + 251 and − 270 bp, seven light response elements (GA, GAG, I-box, TCCC and CHS Unit 1 motifs) located at − 626, + 397,-672, − 551, + 674, + 818 and + 192 bp, a meristem-specific regulatory element (CAT-box) located at − 471 bp, a MYBHv1 binding site (CCAAT-box) located at − 761 bp, a gibberellin (GA) response element (GARE motif) located at + 599 bp, and four endosperm expression elements (GCN4 and Skn-1 motifs) located at + 34, − 250, − 94 and − 88 bp. One MYB binding site involved in drought control is located at + 586 bp, and a corn protein metabolism regulation element (O2 site) is located at + 87 bp. One seed-specific expression regulatory element (RY element) is located at + 329 bp, an SA response element (TCA element) is present at − 395 bp, and a cis-acting element associated with circadian rhythms is located at − 234 bp. Both *BpMYB21* and *BpMYB61* promoter sequences possess MYB transcription factor binding MBS components and bHLH TF binding site G-box components, indicating interactions between the MYB and bHLH TFs. The presence of these regulatory elements indicates the expression of *BpMYB21* and *BpMYB61* in birch in response to a variety of plant hormones and adverse conditions.

**Fig. 5:**
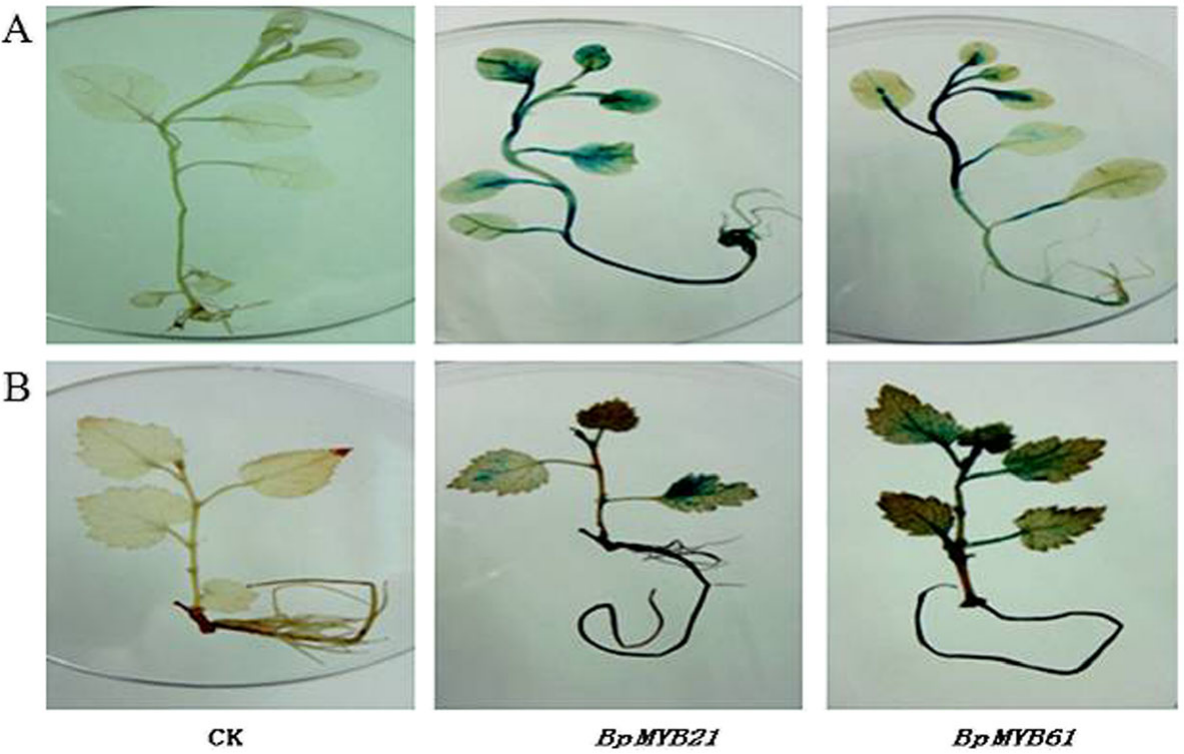
The activity of BpMYB21 and BpMYB61 promoters. CK indicates wild type Tobacco or birch. **a** Tissue culture tobacco plantlets. **b** Tissue culture birch plantlets

The promoters of both *BpMYB21* and *BpMYB61* possess promoter activity. GUS staining revealed a large area of blue in stems of tobacco and birch infiltrated with PBpMYB61, indicating strong priming activity in stems, but weaker activity in leaves. However, a large area of blue was observed in tobacco leaves/stems and birch leaves infiltrated with PBpMYB21, indicating strong and weak activity in birch leaves and stems, respectively (Fig. 5).

### Spatial and temporal expression patterns of *BpMYB* genes following phytohormone treatment

**Fig. 6:**
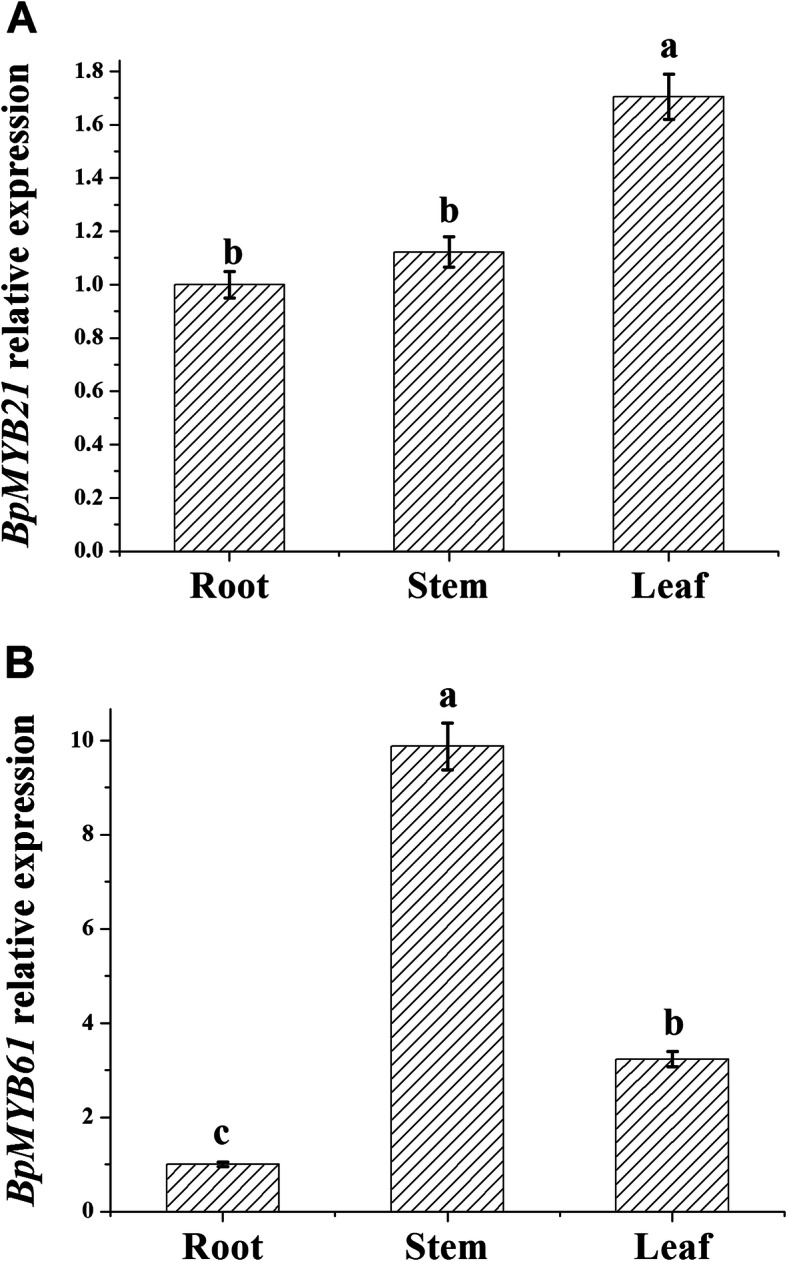
Tissue-specific expression of *BpMYB21* (**a**) and *BpMYB61* (**b**) in birch plantlets. The relative expression of *BpMYB21* and *BpMYB61* was quantified by quantitative RT-PCR. Reported values are means of three replicates, and error bars were obtained from multiple replicates. Values with different superscript letters in a column are significantly different (*p* < 0.05)

**Fig. 7:**
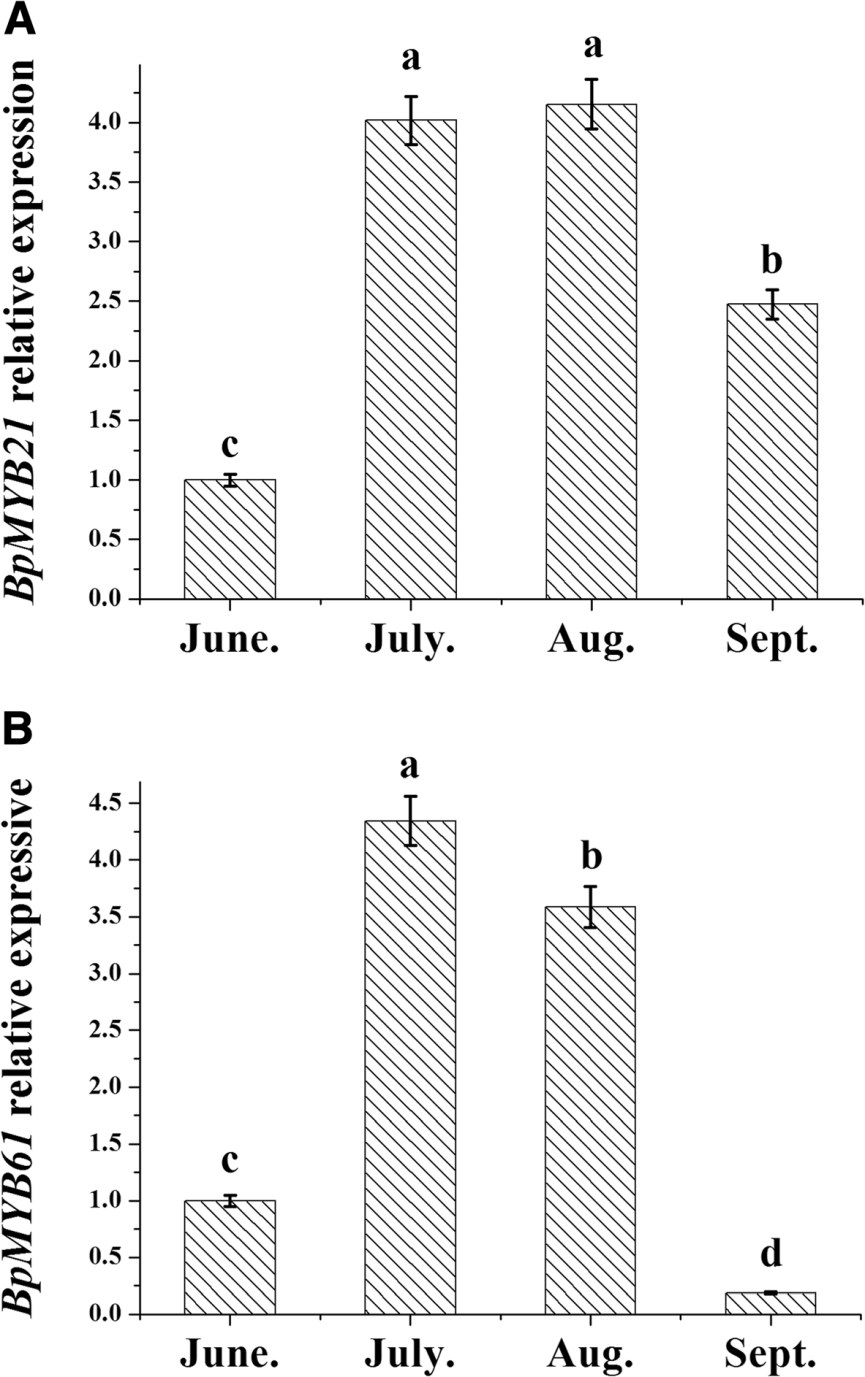
Time-specific expression of *BpMYB21* (**a**) and *BpMYB61* (**b**) in birch plantlets. The relative expression of *BpMYB21* and *BpMYB61* was quantified by quantitative RT-PCR. Reported values are means of three replicates, and error bars were obtained from multiple replicates. Values with different superscript letters in a column are significantly different (*p* < 0.05)

Different tissue expression patterns were observed for the two *BpMYBs* (Fig. 6). *BpMYB21* was highly expressed in leaves, followed by stems, and minimal expression in roots, while *BpMYB61* was highly expressed in stems, followed by leaves, and expression was again lowest in roots. Differences in temporal aspects of expression were also observed (Fig. 7); expression of both *BpMYB21* and *BpMYB61* was significantly higher in July and August.

**Fig. 8:**
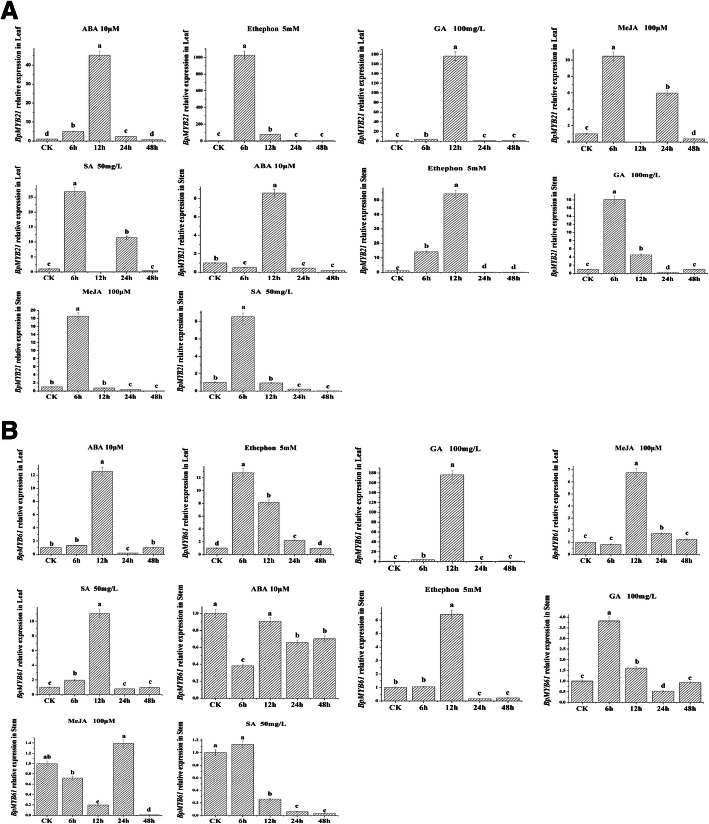
**a**, **b**, **c**, **d** Relative expression of *BpMYB21* and *BpMYB61* in birch treated by different hormones. **a** and **b** relative expression of *BpMYB21* in leaf and stem tissue of birch saplings treated by different hormones, respectively. **c** and **d** relative expression of *BpMYB21* in leaf and stem tissue of birch saplings treated by different hormones, respectively. The relative expression of *BpMYB21* and *BpMYB61* was quantified by quantitative RT-PCR. Reported values are the means of three replicates. Values with different superscript letters in a column are significantly different (*p* < 0.05)

Differences in expression patterns were also observed following treatment with various hormones (Fig. 8a, b). In leaves, expression of *BpMYB21* was significantly increased after treatment with MeJA, SA or ethylene for 6 h, and ABA or GA for 12 h. In stems, expression of *BpMYB21* was also significantly increased after treatment with Me JA, SA or GA for 6 h, and ABA or ethephon for 12 h (Fig. 8a). Similarly, expression of *BpMYB61* was also significantly increased in leaves after treatment with these hormones. Expression of *BpMYB61* was up-regulated after treatment with ABA, Me JA, SA or GA for 12 h, and ethephon for 6 h, compared with the control group. However, expression patterns of *BpMYB61* in stems obtained after treatment with these hormones were different from those observed for leaves*.* Expression of *BpMYB61* transcripts was significantly decreased after ABA, MeJA or SA treatment for 48 h (Fig. 8b). Interestingly, expression of *BpMYB61* was increased 3.82- and 6.42-fold after GA treatment for 6 h, and ethephon for 12 h, compared with controls. These results suggest that *BpMYB21* and *BpMYB61* respond differently to hormone induction, which is also tissue-specific.

### Subcellular localisation of BpMYB21 and BpMYB61

**Fig. 9:**
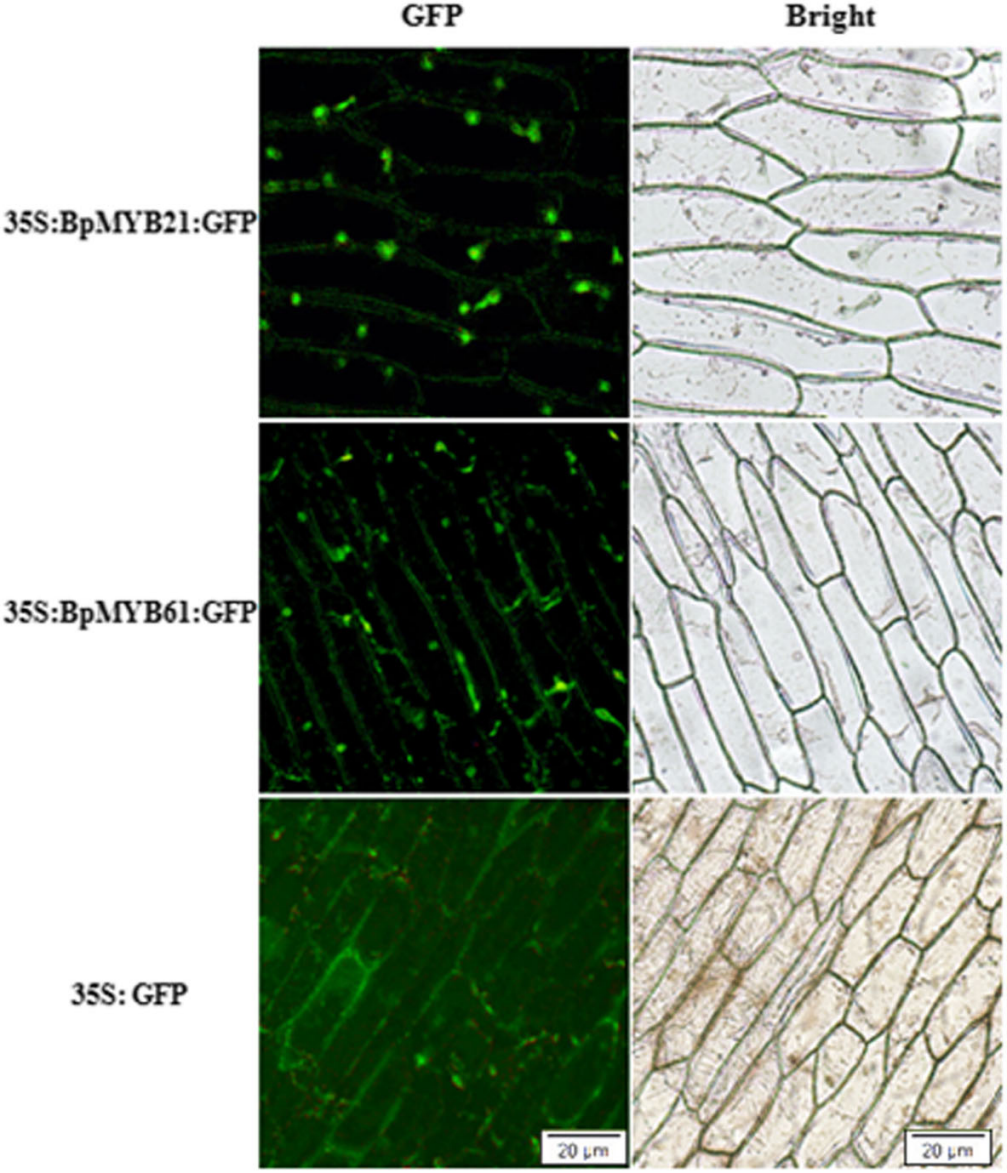
Subcellular localisation of BpMYB21/GFP and BpMYB61/GFP fusion proteins in onion epidermal cells

We investigated the subcellular localisation of BpMYB21 and BpMYB61 in living cells using transient expression assays in onion epidermal cells with constructs expressing green fluorescent protein (GFP), BpMYB21/GFP, or BpMYB61/GFP fusions. Fluorescence microscopy showed that BpMYB21/GFP was present in nuclei, while BpMYB61/GFP was targeted in nuclei and cell membranes (Fig. 9). The GFP control was distributed throughout the cell (Fig. 9). From the typical optical microscope image (Fig. 9, right panel), we can clearly see the position of the onion epidermal nucleus. Therefore, co-localisation of specific proteins in the nucleus was not detected in this experiment.

### Analysis of squalene and total triterpenoid accumulation in INVScl transformants

**Fig. 10:**
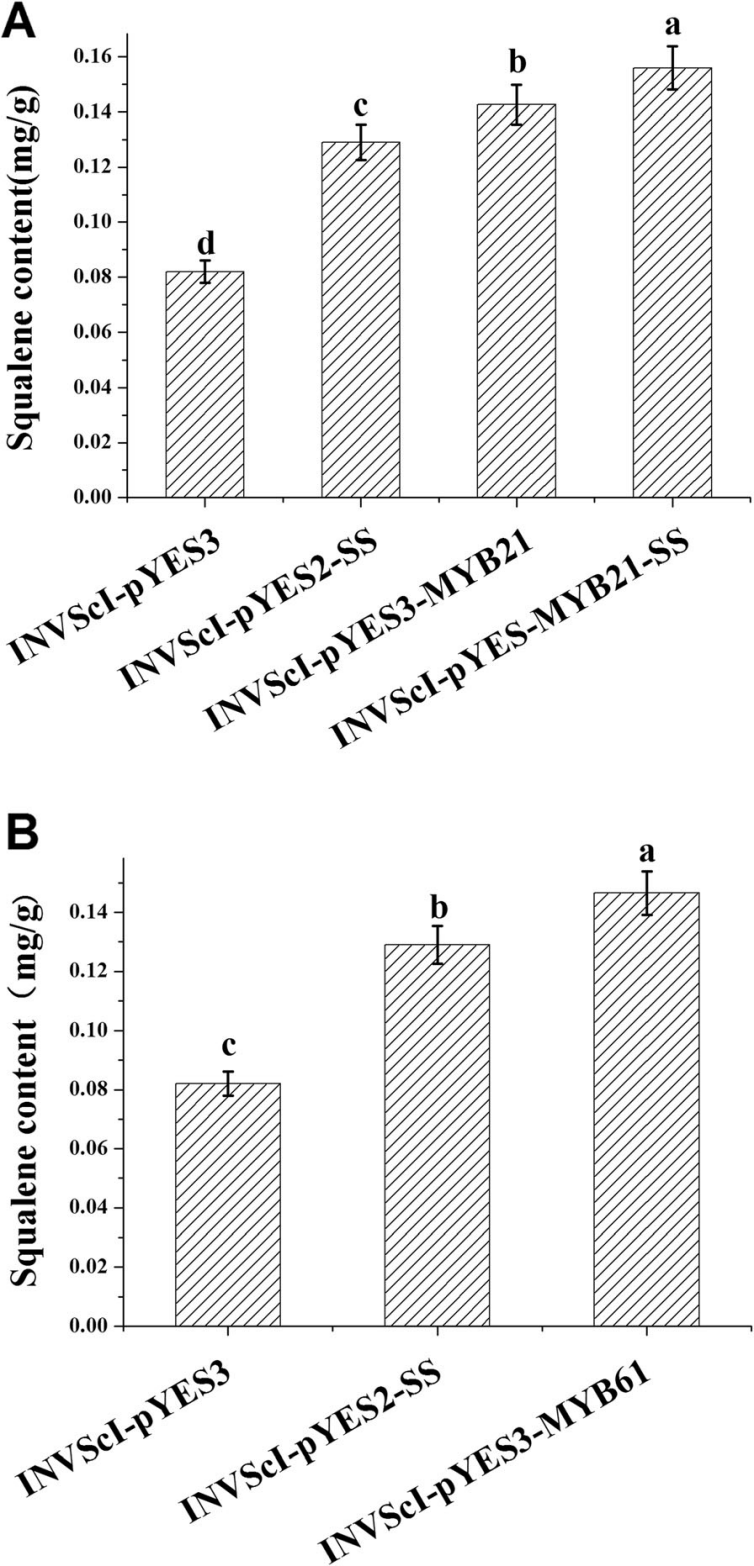
**a** and **b** Squalene content in transgenic yeast cells. A and B indicate the squalene content of recombinant INVSCI-pYES-MYB21-SS and INVSCI-pYES3-MYB61 strains, respectively. The reported values are means of three replicates. Values with different superscript letters in a column are significantly different (*p* < 0.05)

To study the functions of BpMYB21 and BpMYB61, *BpMYB21* and *BpMYB61* genes were incorporated into INVScl and INVScl-pYES2-SS competent yeast cells and terpene synthesis was investigated. Recombinant and control yeast were induced by galactose for 12 h, and the squalene synthase product was extracted and analysed by high-performance liquid chromatography (HPLC) [26]. After culturing for 12 h, the squalene content in INVScl-pYES-MYB21-SS cells (0.155 mg/g) was increased by 89% compared with control yeast cells harbouring empty vector, 20% higher than in INVScl-pYES2-SS cells and 9% higher than in INVScl-pYES3-MYB21 cells (Fig. 10). The squalene content in INVScl-pYES3-MYB61 cells was 0.146 mg/g, 78% higher than in empty vector control yeast cells, and 13% higher than in INVScl-pYES2-SS cells (Fig. 10).

**Fig. 11:**
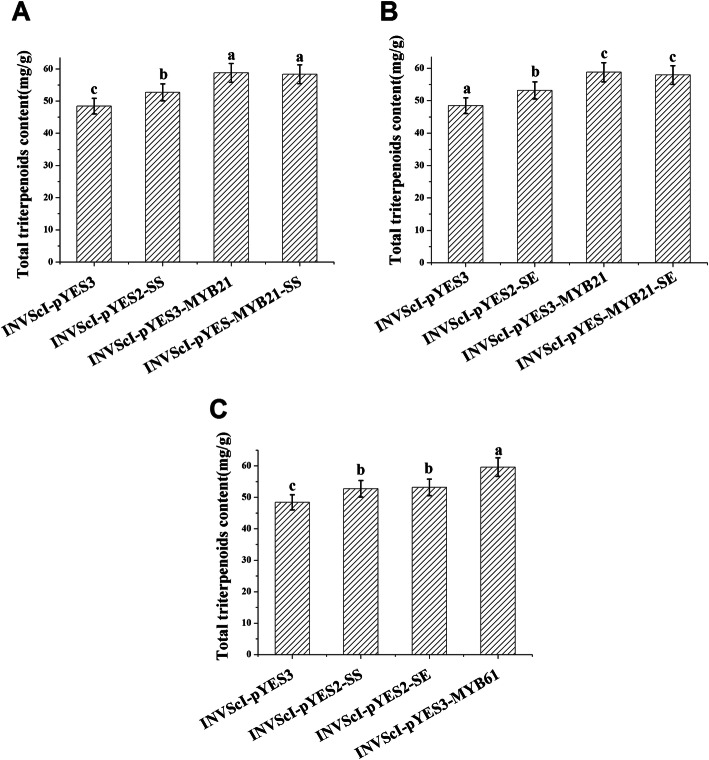
**a**, **b**, **c** Total triterpenoid content in transgenic yeast strains. **a** and **b** indicate the total triterpenoid content of recombinant INVSCI-pYES-MYB21-SS and INVSCI-pYES-MYB21-SE strains, respectively; **c** Total triterpenoid content of INVSCI-pYES3-MYB61 recombinant yeast. The reported values are the means of three replicates. Values with different superscript letters in a column are significantly different (*p* < 0.05)

To further study the functions of BpMYB21 and BpMYB61, recombinant and control yeasts were inoculated for 12 h, followed by extraction of triterpenoids and determination of total triterpenoid content [10]. The total triterpenoid content in INVScl-pYES-MYB21-SS cells was 58.333 mg/g, 20% higher than in control yeast cells harbouring empty vector, and 10% higher than in INVScl-pYES2-SS cells. The total triterpenoid content in INVScl-pYES3-MYB21 cells was increased by 21% compared with control yeast cells. The total triterpenoid content in INVScl-pYES3-MYB61 cells was 59.625 mg/g, 23% higher than control yeast cells, and 13% higher than in INVScl-pYES2-SS cells (Fig. 11). These results indicate that BpMYB21 and BpMYB61 are involved in the synthesis of squalene and triterpenoids.

### Expression of genes related to triterpene synthesis in transgenic birch plantlets

**Fig. 12:**
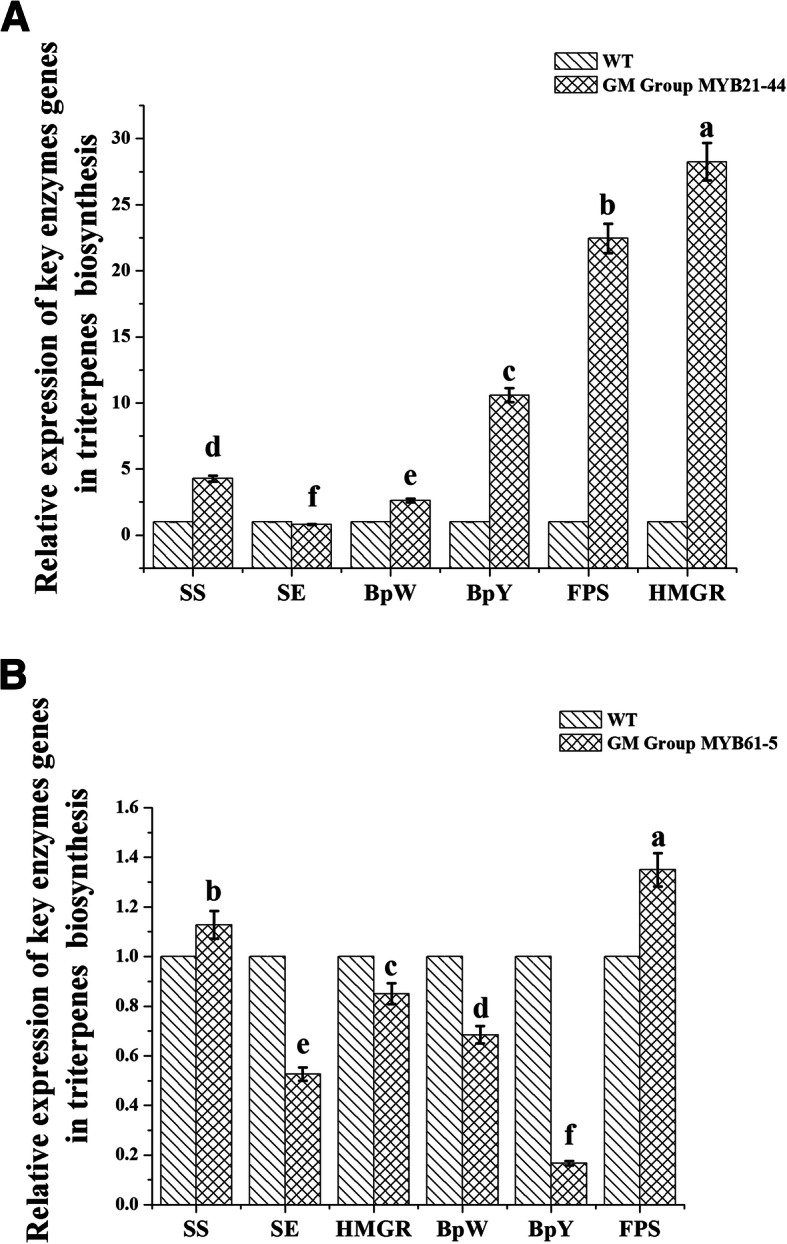
**a**, **b** Relative expression of key enzymes in the MVA pathway in *BpMYB21* and *BpMYB61* transgenic birch plantlets. **a** and **b** indicate the relative expression of key enzymes (*SS*, *SE*, *BPY*, *BPW*, *FPS* and *HMGR*) in the MVA pathway in *BpMYB21* and *BpMYB61* transgenic birch plantlets, respectively. The reported values are the means of three replicates. Values with different superscript letters in a column are significantly different (*p* < 0.05)

To study the role of *BpMYB21* and *BpMYB61* genes in triterpenoid synthesis in birch, we generated transgenic plantlets. Expression of genes related to triterpene synthesis (*SS*, *SE*, *BPY*, *BPW*, *FPS* and *HMGR*) in transgenic plants (lines MYB21–44 and MYB61–5) overexpressing *BpMYB21* or *BpMYB61* was measured using real-time RT-PCR. In MYB21–44 transgenic plants, expression of *SS*, *BPW*, *BPY*, *FPS* and *HMGR* was up-regulated to varying degrees, by 4.27-, 2.61-, 10.59-, 22.44- and 28.24-fold compared with wild birch (controls), respectively (Fig. 12a). Among these, *HMGR* underwent the most significant up-regulation. Expression of *SE* was 79% that in wild birch, representing a slight down-regulation. These results suggest that *BpMYB21* may positively regulate the expression of *SS*, *BPW*, *BPY*, *FPS* and *HMGR* genes in birch.

In MYB61–5 transgenic plants, expression of *SS* and *FPS* showed a similar degree of up-regulation, by 13 and 35%, respectively, compared with wild birch. Expression of *HMGR*, *BPW*, *SE* and *BPY* was down-regulated, by 84, 68, 52 and 16%, respectively, and *BPY* was the most significantly altered. These results suggest that *BpMYB61* may negatively regulate the expression of *HMGR*, *BPW*, *SE* and *BPY* genes in birch (Fig. 12b).

### HPLC analysis of betulinic acid, oleanolic acid and betulin in *BpMYB21* and *BpMYB61* transgenic birch

**Fig. 13:**
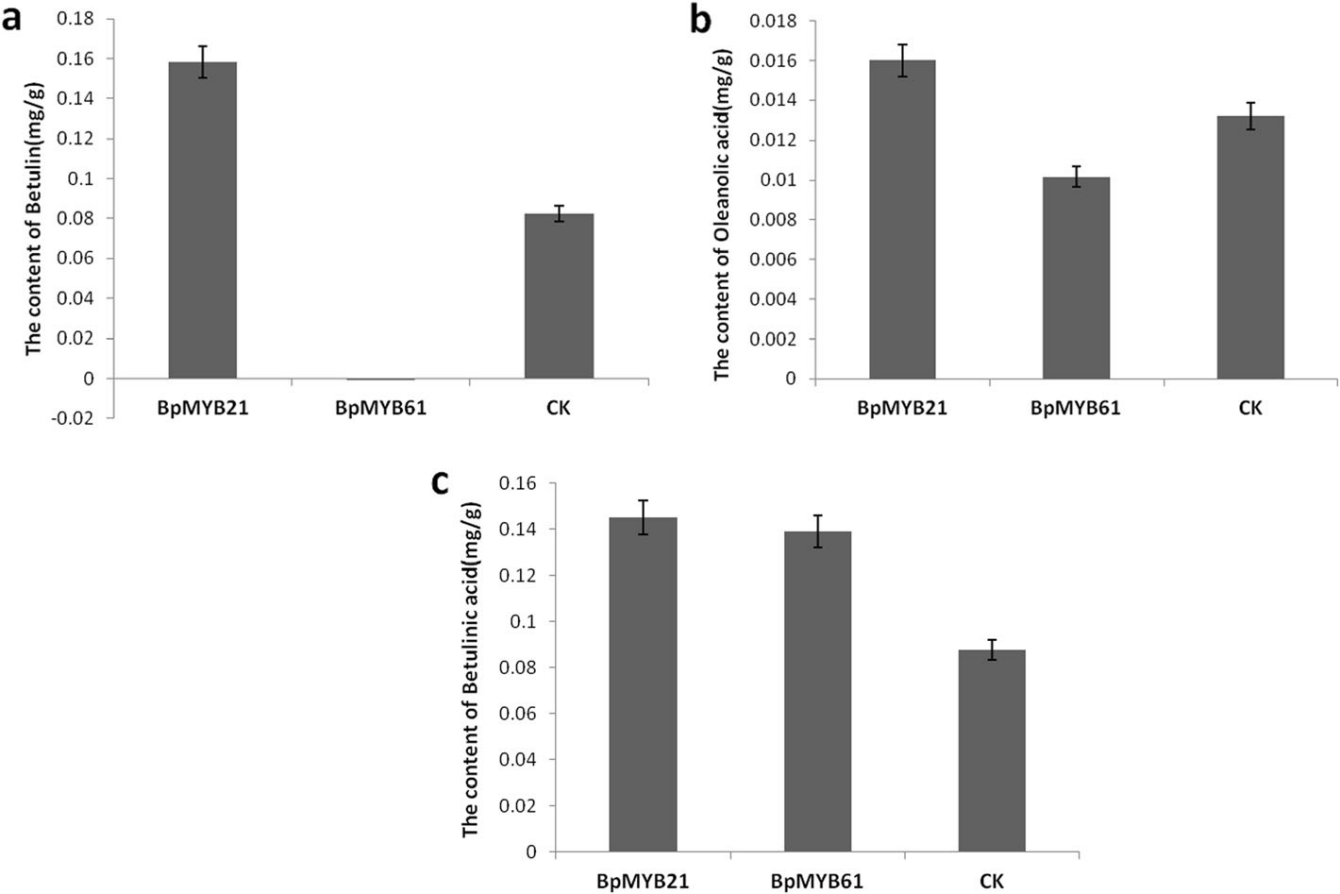
Betulin, oleanolic acid and betulinic acid content in *BpMYB21* and *BpMYB61* transgenic birch. **a** Betulin. **b** Oleanolic acid. **c** Betulinic acid

To further explore the functions of BpMYB21 and BpMYB61 in triterpene synthesis, callus tissue was induced using stem segments of MYB21–44 and MYB61–5 lines greatly overexpressing *BpMYB21* or *BpMYB61*, respectively. Triterpenoids were then extracted, and the content of betulinic acid, oleanolic acid, and betulin in callus tissue was determined using standard curves and regression equations y1 = 9 × 106x + 68,020 (R2 = 0.9994), y2 = 107x + 43,301 (R2 = 0.9993), and y3 = 107x + 63,763 (R2 = 0.9995), respectively. The results showed that the content all three molecules in callus tissue was higher in the MYB21–44 line than in wild birch. Compared with controls, the content of betulinic acid, oleanolic acid and betulin in MYB21–44 transgenic callus tissue was increased by 65, 21 and 92%, respectively. In MYB61–5 transgenic birch, the content of betulinic acid was increased by 59%, but oleanolic acid and betulin were decreased by 23 and 92%, respectively, compared with wild birch. Among the three tested compounds, overexpression of *BpMYB21* increased the content of betulin the most, followed by betulinic acid and oleanolic acid. Overexpression of *BpMYB61* decreased the content of betulin the most, followed by oleanolic acid (Fig. 13). These results further indicate that BpMYB21 and BpMYB61 are involved in the synthesis of triterpenoids.

### Regulation of triterpene biosynthesis genes in birch by BpMYB21 and BpMYB61 transcription factors

**Fig. 14:**
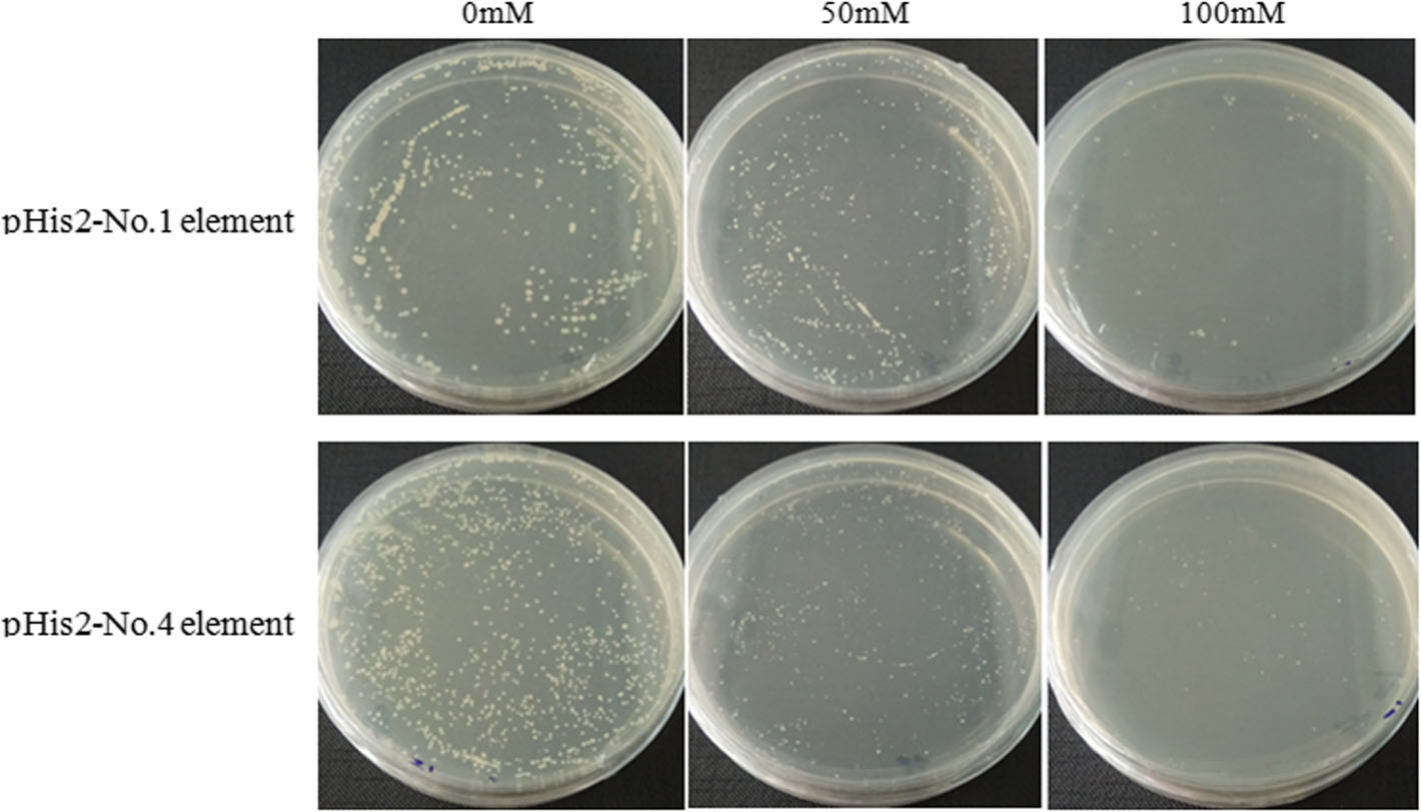
Self-activation detection of pHis2-element recombinant vectors. Transcriptional autoactivation of Y187 recombinant yeast for element No.1 and No.4 was screened on SD/−His/−Trp (DDO) medium with the inhibitor 3-amino-1, 2,4-triazole (3-AT) at 0–100 mM

**Fig. 15:**
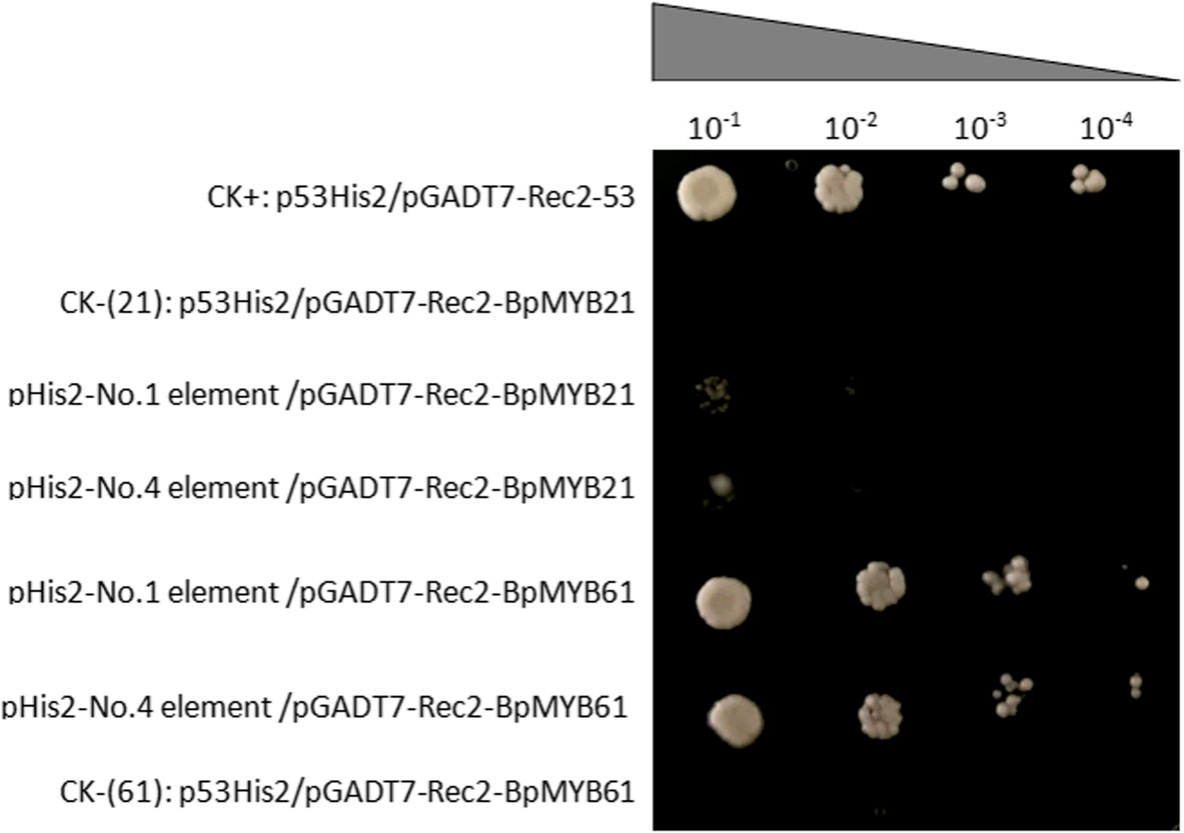
Interaction of MBS elements with BpMYB21 and BpMYB61. Binding of BpMYB21 and BpMYB61 to MBS by Y187 was determined. Three to five tandem copies of the MBS sequence were cloned separately into pHIS2 as reporter constructs. Transformants were screened on SD/−His/−Leu/−Trp (TDO) medium with 100 mM 3-amino-1,2,4-triazole (3-AT). p53HIS2/pGADT7-p53 and p53HIS2/pGADT7-BpMYB21 (and p53HIS2/p GADT7-BpMYB61) served as positive and negative controls, respectively. Triangles indicate the yeast dilutions (1:1, 1:10, 1:100 and 1:1000)

Regulation of *SE* and *BPX* genes by BpMYB21 and BpMYB61 TFs was verified by yeast one-hybrid assays. Specifically, we screened six types of MYB binding elements and successfully constructed three copies of concatenated cis-acting element bait and prey vectors containing *BpMYB21* and *BpMYB61*, respectively. Self-activation results showed that transcriptional activation of elements 1 and 4 could be inhibited by 100 mmol/L 3-AT (3-amino-1,2,4-triazole), while 2, 3, 5 and 6 were not inhibited (Fig. 14). This could be because in the absence of a bait, there could be an endogenous regulatory factor in yeast that may identify and bind the target sequence, making it incompatible with yeast one-hybrid screening. These results indicate a weak interaction between *BpMYB21* and elements 1 and 4 in the promoters of the *SE* and *BPX* genes. By contrast, there was a strong interaction between *BpMYB61* and elements 1 and 4, which indicates that *BpMYB61* has a potent regulatory effect on *SE* and *BPX* genes (Fig. 15).

## Discussion

MYB TFs regulate the synthesis of plant secondary metabolites, and play an important role in flavonoid metabolism [27], anthocyanin biosynthesis, and other secondary metabolite in *A. thaliana* [27, 28]. For example, overexpression of AtMYB75 increases the accumulation of secondary metabolites such as anthocyanins and flavonols [29], while overexpression of AtMYB3, AtMYB6 and AtMYBL2 inhibits anthocyanin biosynthesis in this species [30]. Meanwhile, the WD40-bHLH-MYB complex also regulates plant physiological and biochemical responses to some extent. For example, plant hormones act upstream to induce MYB-bHLH-WD40 complexes, thereby regulating the formation of glandular hairs in *Artemisia annua* [31].

In the present study, we first cloned full-length *BpMYB21* and *BpMYB61* TFs from *B. platyphylla* Suk. using RT-PCR, and temporarily named them *BpMYB21* and *BpMYB61*, respectively. NCBI BLAST alignment analysis revealed ~ 64% amino acid sequence homology between *BpMYB21* and MYBs from *Juglans regia*, *Populus euphratica* and *Vitis vinifera*. Conserved domain analysis revealed one Myb DNA-binding and two SANT-specific motifs (Fig. 1). Similarly, *BpMYB61* was found to be 61–97% homologous to MYBs from *Populus euphratica*, *Juglans regia*, *Populus trichocarpa* and *Betula luminifera*. The polypeptide sequence encoded by *BpMYB61* also contains a Myb DNA-binding motif. Thus, we propose that *BpMYB21* and *BpMYB61* are novel R2R3-MYB genes in birch.

We obtained the promoters of *BpMYB21* and *BpMYB61* from *B. platyphylla* using a genome walking approach, yielding 1302 and 850 bp fragments, respectively. The *BpMYB21* and *BpMYB61* promoters contain many typical components including TATA and CAAT boxes, as well as stress-related cis-elements that facilitate adaptation to adverse environmental conditions. The promoters also contain plant hormone response elements such as ABRE, CGTCA, TCA and GARE motifs, indicating that *BpMYB21* and *BpMYB61* are regulated by plant hormones. Similarly, AtMYB30 and BES1, AtMYB77 and ARF7, and AtMYB18 and FHY1FHL are involved in the regulation of brassinolides, auxins and optical signals in Arabidopsis [32, 33]. Analysis of birch transcriptional data revealed that *BpMYB21* and *BpMYB61* genes may be induced by MeJA and SA signals, and *BpMYB21* and *BpMYB61* were up- and down-regulated by MeJA and SA, respectively. Additionally, expression of *BpMYB21* and *BpMYB61* was induced by different hormones. The MYB gene family is usually divided into four subfamilies according to the number of MYB repeats in the MYB domain, namely R1R2R3-MYB (3R-MYB), R2R3-MYB (2R-MYB), and 4R-MYB, containing three, two, and four MYB repeats, respectively; and the majority of MYB TFs in plants are either R2R3-MYB or R1-MYB types [28, 34]. In the present study, bioinformatics analysis showed that BpMYB21 and BpMYB61 are both R2R3-MYB family proteins (Figs. 1 and 3).

Hormones such as JA, SA and ethylene are involved in signal transduction related to plant secondary metabolism, forming a complex regulatory network [35]. For example, in the JA signal transduction pathway, the repressor jasmonate ZIM-domain (JAZ) that is involved in the flavonoid metabolic pathway acts as a direct inhibitor and MYB TF, promoting the regulation of target genes by MYB TFs, thereby affecting the metabolism of flavonoids [36]. Exogenous SA promotes the synthesis of secondary metabolites such as terpenes, alkaloids and flavonoid compounds [37–39]. In a previous study, we demonstrated that MeJA and SA significantly enhance the content of triterpenes in birch, and up-regulate the expressions of genes involved in triterpene biosynthesis [23–25]. In the present study, *BpMYB21*- and *BpMYB61*-expressing transgenic plants were treated with ABA, GA, Me JA and SA, and responses were elevated compared with controls, indicating that expression of *BpMYB21* and *BpMYB61* is indeed affected by plant hormone induction. The *BpMYB21* gene displayed expression patterns consistent with MeJA and SA, displaying responses within 6 h in stems and leaves. Expression patterns in leaves and stems in response to ABA showed a similar trend, with up-regulation after 12 h. The *BpMYB61* gene was responsive to hormone treatments in stems and leaves for 48 h and 12 h, respectively. There may be a synergistic effect between MeJA and SA on the expression of *BpMYB21*, and the same may be true for ABA, MeJA and SA hormones and *BpMYB61* expression.

In addition, binding sites for MYB TFs such as MBS and bHLH TFs, including G-boxes, were found in the *BpMYB21* and *BpMYB61* promoter sequences. MYB family TFs can not only regulate the synthesis of plant secondary metabolites alone [14]; the same MYB TFs, such as AmMYB305 and AmMYB340, can also regulate the synthesis of secondary metabolites via interactions with each other [40]. For example, bHLH TFs can also regulate the synthesis of secondary metabolites such as glucosides, flavonoids [41] and terpenoids [42], and the biosynthesis of anthocyanins is controlled by the TTG1/bHLH/Myb complex, and the co-regulation efficiency is higher than that achieved by single component action in *Arabidopsis* [43].

Squalene and total triterpenes obtained from yeast are the same as those in plants, and like plants, yeasts can be a good source of these chemicals. Research on the synthesis of terpenoids using yeast expression systems has progressed thanks to the application of synthetic biology to terpenoids. *Escherichia coli* and *Saccharomyces cerevisiae* are often used as hosts for triterpenoid saponin synthesis. Yeast possesses many enzymes and substrates needed for secondary metabolism in plants, and the efficiency of synthesis of secondary metabolites can be much higher than in plants [44]. Indeed, great progress has been made in the synthesis of terpenoids using yeast expression systems and synthetic biology approaches [45]. For instance, the genetic stability of an engineered strain was improved by integrating the mevalonate (MEV) pathway into the yeast genome, overexpression of the *Amorpha fruticosa* 411-diene synthase gene in *Artemisia* was enhanced 500-fold, and the yield of the sesquiterpenoid artemisinin was increased [46]. In another study, using yeast as a host, UDP-glycosyltransferase, CYP716A47, and its coenzyme ATR2–1 from *Panax ginseng* were co-expressed, and triterpenoids including panaxadiol saponins CK could be synthesised from scratch [47]. In the present study, the squalene content and total triterpene content in INVScl-pYES3-MYB21 and INVScl-pYES3-MYB21-SS recombinant yeast cells were increased to different degrees. The squalene content of INVScl-pYES-MYB21-SS yeast cells was increased by ~ 89% compared with control yeast cells, and the squalene and total triterpene content in INVScl-pYES3-MYB61 recombinant yeast were increased in the order INVScl-pYES3-MYB61 > INVScl-pYES2-SS > INVScl-pYES3. These results suggest that *BpMYB21* and *BpMYB61* function in the synthesis of squalene and triterpenes, but the extent of improvement was limited. Expression of *SS*, *BPY*, *BPW*, *FPS* and *HMGR* was up-regulated significantly in transgenic birch overexpressing *BpMYB21*. Expression of *SS* and *FPS* were similarly up-regulated, and expression of *HMGR*, *BPW*, *SE* and *BPY* were comparably down-regulated. Meanwhile, changes in triterpene product content in MYB21–44 and MYB61–5 transgenic lines was similar to that resulting from MVA pathway gene expression. Expression of MVA genes and the yield of triterpenoid products were up-regulated in BpMYB21–44 lines, and down-regulated in BpMYB61–5 lines. Although the content of triterpenoid products in MYB61 yeast was increased, the increase was very small compared with that in MYB21 yeast. In addition, in previous studies, we obtained the promoter sequences of key genes involved in triterpene synthesis (unpublished), and found that the promoters of the *SE*, *BPW* and *BPX* genes contain cis-acting elements that can bind to MYB TFs. Thus, MYB TFs may regulate the synthesis of triterpenes by binding to cis-acting elements in the promoters of key enzymes in triterpene synthesis. Herein, we confirmed the regulation of *SE* and *BPX* genes in birch by BpMYB21 and BpMYB61 TFs using yeast one-hybrid assays. We have obtained yeast strains containing *SS*/*SE* in our previous studies, and their promoter are GAL1 from yeast, but our results show that when *BpMYB21* and *BpMYB61* enters into yeast strains containing *SS*/*SE*, and the triterpene content is increased. Through our analysis of the GAL1 promoter sequence shown that the GAL1 promoter sequence has a regulatory element of MYB transcription factor, indicating that *BpMYB21* and *BpMYB61* may regulate the expression of *SS*/*SE* genes in yeast, and then regulate triterpene synthesis. At the same time, our previous experiments confirmed that the promoter sequence of *SS* and *SE* genes in birch does contain the MBS sequence of the action site of *BpMYB* transcription factor. It is suggested that *BpMYB21* and *BpMYB61* in birch may regulate the expression of *SS* and *SE* genes, and then regulate triterpene synthesis. We will also pay more attention to the interaction and regulation mechanism of MYB gene and GAL1 promoter in yeast cells in the future.

**Fig. 16:**
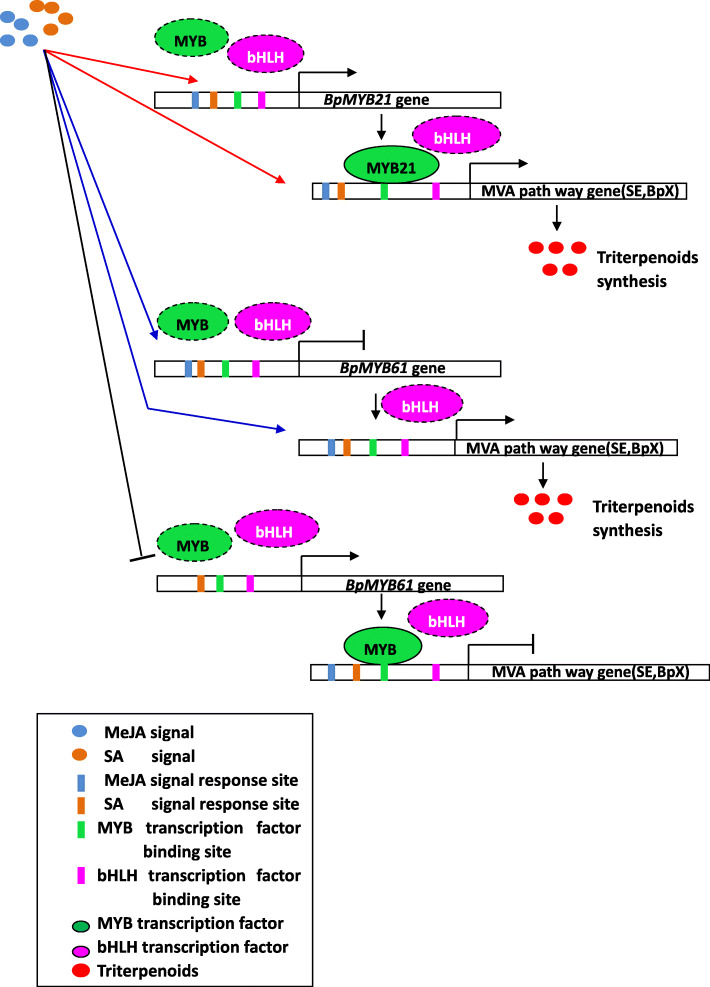
Simplified working model of the role of *BpMYB21* and *BpMYB61* in hormone-induced triterpenoid synthesis in *Betula platyphylla*. *BpMYB21* and *BpMYB61* are induced by SA/MeJA. BpMYB21 and BpMYB61 interact with bHLH to induce *BpSE* and *BpX* expression by binding the MBS elements of the *BpSE* and *BpX* promoter, which subsequently regulates triterpenoid synthesis

However, MYB-acting elements are not the only factors determining whether MYB TFs activate downstream gene promoters; the number and locations of cis-elements and the characteristics of promoter sequences at both ends of the elements can influence the transcriptional activation of promoters [48]. Induction of hormone signals is also a determinant of downstream gene expression. Based on the above results, we deduced that hormone signals such as MeJA or SA may induce the expression of *MYB* genes, and MYB TFs and/or bHLH TFs might also play a regulatory role upstream of *MYB* genes. We speculate that there are two main mechanisms by which MYB TFs regulate terpenoid biosynthesis; by acting directly on relevant cis-acting elements in the promoters of genes encoding key enzymes in terpene synthesis, or by interacting with bHLH TFs; alternatively, MYB TFs may form transcription complexes with bHLH and WD40 TFs to regulate the expression of downstream target genes, and hence the synthesis of terpenoids. However, whether bHLH TFs regulate the expression of *MYB* genes, and whether they form transcription complexes with MYB TFs requires further verification. Additionally, expression of *MYB* genes is regulated by specific genes and external conditions, which also requires further exploration. Based on the results of this study and previous reports, we propose a simplified working model for the role of *BpMYB21* and *BpMYB61* in hormone-induced triterpenoid synthesis in *B. platyphylla* (Fig. 16). In future studies, transgenic plants could be induced by hormones to investigate the regulation of triterpene synthesis by MYB-bHLH transcriptional complexes involved in MeJA or SA signal-inducible responses.

## Conclusion

We cloned *BpMYB21* and *BpMYB61* genes from *Betula platyphylla* Suk. for the first time. *BpMYB21* is a new member of the MYB transcription factor family. *BpMYB21* and *BpMYB61* genes displayed temporal and spatial expression specificity. The expression patterns of these genes were induced by different hormones and injuries, and they were significantly different. The promoters of *BpMYB21* and *BpMYB61* genes possessed promoter activity. *BpMYB21* and *BpMYB61* genes are involved in the synthesis of squalene and triterpenes. In transgenic plantlets, we found that *BpMYB21* and *BpMYB61* genes were involved in the expression of key genes involved in the triterpenoid pathway, but there were significant differences in the regulation of the two genes. BpMYB21 and BpMYB61 had a regulatory effect on *SE* and *BPX* genes, and the regulatory effect of BpMYB61 was stronger. *BpMYB21* and *BpMYB61* genes have great potential for triterpene production and regulation in plants.

## Methods

### Plant material and growth conditions

Plant materials (birch plantlets) used in this study were from our lab (Forest Biological Engineering Laboratory, College of Life Science, Northeast Forestry University, Harbin, China; Professor Yaguang Zhan is the chairwomen of the laboratory, Email: yaguangzhan@126.com). Different tissues from 4-week-old birch plantlets were used in this study. Birch was grown in a growth room under a 16 h:8 h (23–25 °C) light/dark photoperiod as described previously. For treatment with ABA (10 μM), MeJA (100 μM), SA (50 mg/L), ethephon (5 mM), or GA (100 mg/L), birch were completely soaked in WPM liquid medium containing the appropriate hormone solution. Leaves and stems of birch saplings were harvested at different times after phytohormone treatment.

### Gene cloning and sequence analysis

Genomic DNA and total RNA were extracted by the CTAB method. *BpMYB21* and *BpMYB61* gene sequences were derived from birch transcriptome data in our lab. Specific primers (MYB21-full-F/R and MYB61-full-F/R; in Table S1) were designed to amplify the full-length *BpMYB21* and *BpMYB61* genomic sequences and cDNA sequences from birch. PCR products were ligated with the 18-T T-vector (TaKaRa, Dalian, China). Amino acid sequences from different plant species were selected using BLAST software from NCBI. Motif analysis of BpMYBs was performed using the Prosite program (http://prosite.expasy.org/prosite.html) and SMART tool (http://smart.embl-heidelberg.de). The respective domains of MYB proteins were aligned using DNAMAN8.0 (Lynnon Biosoft) and illustrated by Boxshade (http://www.ch.embnet.org/software/BOX_form.html). Alignment of homologous peptide sequences was carried out, and neighbour joining phylogenetic trees were constructed using DNAMAN software. Accession numbers of all sequence data and phylogeny data in Figs. 2 and 4 been provided in Additional file 3.

### Reverse transcription and quantitative real-time PCR

Expression of *BpMYB21* and *BpMYB61* was analysed in root, stem, leaf tissue during different months. Expression of *BpMYB21* and *BpMYB61* was analysed in stem and leaf tissues following treatment with 10 μΜ abscisic acid (ABA), 100 μΜ methyl jasmonate (Me-JA), 50 mM ethephon, 50 mg/L salicyclic acid (SA), and 100 mg/L gibberellins (GAs) for 0, 6, 12, 24 and 48 h. Materials were harvested for RNA extraction, subsequent reverse transcription, and quantitative real-time PCR (qRT-PCR) analysis using RealMasterMix with SYBR Green I (TaKaRa) and an ABI7500 real-time PCR system as described previously [23]. The *TU* gene was used as an internal control. Primers used for reverse transcription and qRT-PCR analysis are listed in Table S2.

### Subcellular localisation

The coding sequences of *BpMYB21* and *BpMYB61* were inserted into the pCAMBIA1303 vector for fusion with green fluorescent protein (GFP) under the control of the 35S promoter to generate the GFP-BpMYB21 and the GFP-BpMYB61 constructs. The fusion constructs pCAMBIA1303-GFP-*BpMYB21* and pCAMBIA1303-GFP-*BpMYB61*, and the control pCAMBIA1303-GFP, were incorporated into strain LBA4404 using the triparental hybridisation method. Subcellular localisation was analysed in onion epidermal cells using agrobacterium-mediated transient expression [49]. Primers used for fusion with GFP are listed in Additional file 3 Table S1. The subcellular distribution of BpMYB21 and BpMYB61 proteins was observed by laser confocal microscopy using an LSM510 Meta UV instrument (Zeiss, Germany). Primers used for *BpMYB21* or *BpMYB61* and GFP fusion are listed in Table S3.

### *BpMYB21* and *BpMYB61* promoter isolation and activity analysis

The promoter regions of *BpMYB21* and *BpMYB61* were isolated by the genome walking method. Genomic DNA was extracted from young birch plants using a DNA extraction kit. Genomic walking PCR was performed with a universal genome walker kit following the manufacturer’s instructions. Primers used in genomic walking PCR, including AP1-AP4 and specific primers, are listed in Table S4. Conserved cis-element motifs located in the *BpMYB21* or *BpMYB61* promoters were identified using PlantCARE (http://bioinformatics.psb.ugent.be/webtools/plantcare/html/). To study the activities of *BpMYB21* and *BpMYB61* promoters, the suicide gene in the pXGUS-P vector was replaced with the *BpMYB21* or *BpMYB61* promoter sequence to generate pMYB21::GUS and pMYB61::GUS. Primers used for fusion with GUS are listed in Table S5. Firstly, the constructed plasmids pMYB21::GUS and pMYB61::GUS were transferred into Agrobacterium strain LBA4404 using the triparental hybridisation method, and whole birch and tobacco plants were cultured with LBA4404 for 48 h to introduce foreign genes into plants. Gus enzyme activity was then tested detect whether the exogenous promoter was active, and to investigated expression location.

### Generation of transgenic plants

In order to verify the functions of *BpMYB21* and *BpMYB61* in controlling triterpene synthesis in birch, they were separately overexpressed in birch plantlets. To generate *BpMYB21* and *BpMYB61* overexpression constructs, coding sequences of *BpMYB21* or *BpMYB61* were inserted into the modified pCAMBIA1303 vector under the control of the 35S promoter. The fusion constructs pCAMBIA1303-*BpMYB21* and pCAMBIA1303-*BpMYB61*, and the control pCAMBIA1303, were incorporated into strain LBA4404 using the triparental hybridisation method. Leaves, petioles and stem segments of birch plantlets served as experimental materials. LBA4404 Agrobacteria containing pCAMBIA1303-BpMYB21 or pCAMBIA1303-BpMYB61 were resuspended in LB culture medium, and infiltrated into leaves, petioles and stem segments of birch plantlets [50]. Callus induction and seedling clumping was performed after removal of Agrobacterium until plantlets had grown to 2 ~ 3 cm using culture conditions as previously described [49]. Primers used to obtain overexpression constructs are listed in Table S6.

### Squalene and triterpenoid analysis in yeast

Coding sequences of *BpMYB21* and *BpMYB61* were inserted into the pYES3 vector. Full-length genes including an *EcoR*I restriction enzyme site were obtained by PCR with primers designed using the Infusion primer design website (https://www.takarabio.com/learning-centers/cloning/in-fusion-cloning-tools), and are listed in Table S7). *BpMYB21* and *BpMYB61* genes were incorporated into the INVScl yeast strain, and INVSc-pYES2-SS yeast cells were analysed for terpenoid synthesis. Yeast transformation was performed using a yeast transformation kit (Cat. No. yp7867-200 t; Beijing Huayue biological Co., Ltd.) following the manufacturer’s instructions. The squalene and total triterpenoid content in *Saccharomyces cerevisiae* InvscI was measured by high-performance liquid chromatography (HPLC) [51]. All experiments were repeated three times.

### Determination of betulinic acid, oleanolic acid and betulin in transgenic birch

The content of betulinic acid and oleanolic acid in transgenic birch was determined by HPLC as previously described [26].

### Yeast one-hybrid assay

Yeast one-hybrid assays were performed using the MATCHMAKER one-hybrid system. Fragments of 3–5× MBS motifs were synthesised by oligonucleotide annealing and ligated into the *EcoR*I site of the pHis2 vector. Bait constructs were linearised with *Sma*I and integrated into the yeast genome (strain Y187). Various concentrations of 3-AT on SD/−T/−H medium were used to measure the basal expression of HIS. *BpMYB21* and *BpMYB61* ORFs were ligated into the GAL4 activation domain of pGADT7-Rec2, and yeast transformants were tested on SD/−T/−H/−L medium containing 100 mM 3-AT. Primers used for bait and prey constructs are listed in Table S8 and in Table S9.

## Supplementary information

**Additional file 1. **Nucleotide sequence and deduced amino acid sequence of *BpMYB21* from birch.

**Additional file 2. **Nucleotide sequence and deduced amino acid sequence of *BpMYB61* from birch.

**Additional file 3.** Accession numbers in NCBI of all sequence data and phylogeny data in Figs. 2 and 4.

**Additional file 4. **Sequences of *BpMYB21* and *BpMYB61* promoters from birch.

**Additional file 5: Table S1.** Specific primers used for gene cloning and sequence analysis.

**Additional file 6: Table S2.** Primers used for reverse transcription and quantitative real-time PCR (qRT-PCR) analysis.

**Additional file 7: Table S3.** BpMYB21, BpMYB61 and GFP fusion primer design for subcellular localisation analysis.

**Additional file 8: Table S4.** Intron primers for BpMYB21 and BpMYB61

**Additional file 9: Table S5.** Specific primers for BpMYB21 and BpMYB61 promoter cloning.

**Additional file 10: Table S6.** Primers for promoter activity analysis

**Additional file 11: Table S7.** Primers for construction of yeast expression vectors for squalene and total triterpenoid analysis.

**Additional file 12: Table S8.** Primers for component synthesis and construction of the bait vector for yeast one-hybrid assays.

**Additional file 13: Table S9.** Primers for construction of the prey vector for yeast one-hybrid assays.

## Data Availability

All supporting data are included as additional files. The datasets generated and/or analysed during the current study are available in the [PERSISTENT WEB LINK OR ACCESSION NUMBER TO DATASETS] repository.

## Abbreviations

FPS: Farnesyl pyrophosphate synthase
SS: Squalene synthase
HMGR: 3-hydroxy-3-methylglutaryl coenzyme a reductase
BPW: Lupeol synthase
SE: Squalene epoxidase
BPY: β-amyrin synthase
BPX: Cycloartenol synthase
ABA: Abscisic acid
GA: Gibberellin
MeJA: Methyl jasmonate
SA: Salicylic acid
HPLC: High-performance liquid chromatography
SD/−T: Synthetic Dropout Medium lacking Trp
SD/−H: Synthetic Dropout Medium lacking His
SD/−L: Synthetic Dropout Medium lacking Leu
