# Supplementary material for: Functional identification of BpMYB21 and BpMYB61 transcription factors responding to MeJA and SA in birch triterpenoid synthesis
Source: BMC Plant Biol. 2020 Aug 12;20:374. doi: 10.1186/s12870-020-02521-1 (PMC7422618; doi:10.1186/s12870-020-02521-1)
Supplement: Supplementary file 1 — Additional file 1. Nucleotide sequence and deduced amino acid sequence of BpMYB21 from birch. [file 12870_2020_2521_MOESM1_ESM.pdf]

Nucleotide sequence of BpMYB21 from birch

TCGCCATCATCCTCTCTCTCTCTCACTTTCTCTCCGTCTCTATCTGTCGTCTCACACAAACACACTCTT
CTATCAAAGTATTGATCACAAGTTTTGTGAAGATACAATGGGAAAATCTCCTTGTTGTGAAAAAGATGG
AATAAAGAAAGGTCCATGGACTGCTGAGGAAGATCAGAAACTGGTTGATTATATTCAAAAGCATGGACA
CGGTAGATGGCGAATCCTTCCTAAGAATGCAGGTTTGAAAAGGTGTGGAAAGAGCTGCCGGCTTCGCTG
GACAAACTATTTGAGACCTGACATCAAGAGAGGGAGGTTTTCGTATGAAGAAGAAGAGACCATAATACA
ACTGCACGGTGTTTTGGGAAATAAGTGGTCCGCTATTGCTGCTCACTTGCCAGGAAGAACAGATAATGA
GATTAAGAACTATTGGAACACCCACATCAGAAAAAGGCTACTAAGAATGGGAATTGATCCAGTGACTCA
CACCCCACGCCTTGATCTTCTCGAACTCTACTCAATTCTGAACTCATCTCAACTTAATCTCCCCAGTTT
GCTAGGGATTGGACCTATTGTCAACCCGAATCTCCTGAGTCTAGCCACAGCTCTCTTGTCATCCCAATG
CAAAAACCCCGATACTAATTCTCAATATCTTCAGCAAAACCAACTTGATGACAATATTCCAATTGAAAA
CCAGTTCCAGTCCACCCAACCAATTCAGCCAAACACAACTTATTTCTCACAAGCACCACTACTAAACTC
TCTAAGCACTCCCTTTCAAAGTGAAACCCAGCTGACAGATCAACCCAAAATGGAGCAAATCTCTCCAGA
TTTTACCAATTTTAGCTGCCAAAATTCTGTACCAAATTTATGGCAAAATAACGGACAGAATCCTGCAGG
TTTAGCTATGAGCGATATTCTGTACCATGATCAGCGCGTACCAAATTTCAGCTTTGGCTCTCTTATTTC
GACAGATTCATCTAGCGATCCCACTCCCCTAAATTCATCTTCTACTATCACCTATGCCAACTGTAGCAC
TGAAGATGAGAGGGATAGTTACTGCAGCAACATTTTGATGTATGATATGCCAAATAGCTTGACTGCCCG
TGGATTGCATATGTAA

Amino acid sequence of ORF of BpMYB21 from birch

MGKSPCCEKDGIKKGPWTAEEDQKLVDYIQKHGHGRWRILPKNAGLKRCGKSCRLRWTNYLRPDIKRGR
FSYEEEETIIQLHGVLGNKWSAIAAHLPGRTDNEIKNYWNTHIRKRLLRMGIDPVTHTPRLDLLELYSI
LNSSQLNLPSLLGIGPIVNPNLLSLATALLSSQCKNPDTNSQYLQQNQLDDNIPIENQFQSTQPIQPNT
TYFSQAPLLNSLSTPFQSETQLTDQPKMEQISPDFTNFSCQNSVPNLWQNNGQNPAGLAMSDILYHDQR
VPNFSFGSLISTDSSSDPTPLNSSSTITYANCSTEDERDSYCSNILMYDMPNSLTARGLHM
