# Supplementary material for: Functional identification of BpMYB21 and BpMYB61 transcription factors responding to MeJA and SA in birch triterpenoid synthesis
Source: BMC Plant Biol. 2020 Aug 12;20:374. doi: 10.1186/s12870-020-02521-1 (PMC7422618; doi:10.1186/s12870-020-02521-1)
Supplement: Supplementary file 2 — Additional file 2. Nucleotide sequence and deduced amino acid sequence of BpMYB61 from birch. [file 12870_2020_2521_MOESM2_ESM.pdf]

Nucleotide sequence of BpMYB61 from birch

TCTCTCTCTCTCTCTCTCTAAACATCTTTTCTACTCCACAACTCCCTTAAAAATCCATTTCAGAGCCAC
CTCTAGCCGTTGCTGACAGTTCTCCTAGGATTCAACAAAATTGCTGCTGTTTTAAGGATAGCTTCTCCC
TCATAGCAGGTGGGTCTTGCTCTAAAATGGGGAGGCACTCTTGCTGTTACAAGCAGAAGCTAAGGAAAG
GCCTTTGGTCACCAGAGGAGGATGAGAAGCTTCTGAGGCATATTACCAAGTATGGTCATGGCTGTTGGA
GCTCTGTTCCTAAGCAAGCCGGTCTGCAGAGGTGTGGGAAGAGCTGCAGATTGAGGTGGATCAACTACT
TGAGGCCTGATTTGAAGAGAGGTACATTCTCGCAGGAGGAAGAGAATCTCATAATCGAACTTCATGCAG
TTCTCGGGAACAGGTGGTCTCAGATTGCAGCACAATTGCCTGGAAGAACCGACAATGAAATAAAAAATC
TATGGAACTCTTGCTTGAAGAAGAAGCTGAGGCAGAGAGGTATTGACCCCGTCACCCACAAACCAATTT
CCGAAGTGGAGAACGGAGAGGAGGATAAAGATACAGAAACCAAAAGCAAAGACAAAATGTCGGTGGTAT
CCAATGAACTCAATCTCCTCAAGGCAGAAAGTTCAAGGCAAGAATCCCCAATTGCTGCACAAGGCTACC
GATTGGAAGTGCAGGGTAACAATTTGATGACACCCACAGCTAACAATGATTTCCTCCACCAAGATACTT
CCACAACCAACTGCCAGCCGTCAGAGTTGGTGGGACATTTTCCGCTTCAGCAATTGAATTATGTATCCA
ATGCCACAAACTCCAACCCCACGCAGTGGTTCACCCAAAGTGGGAAATCGTTTGATATGAATTCTGAGT
TTGCCTCCATGCTACCAAACTCTATTGGTTACAAGCCTTCGCTTACTTTTCCCGCCGACAATGTATCCA
TGAGCTCTTTCACAGTGAATGGATCCCGTTACTGGGAAGGCAGTGGCGCCGCCGCCACCAACAGCAACA
GCGGAAGCAGCAGCAGCGCTGAGTTGCAAAGCAACAGCTCCTTGCTGGAGAACAGCATGTTTTCGTGGG
GATTGGCTGATTGTGGCACATCAGATAAGGAGACCCAAACCCATCTGACCGAAGACATCAAGTGGGCAG
AATATTTTAATAACCCATTATTGATGGCGGCTGCTTTACAAAATCAAACACCACAGTGTTTATACAATG
AGATAAAATCAGAAACGCATTTGGTGACCGATACTTCAACTGCTATGTGGGCACATCATAACAAGCAGC
AGGGATCTTTGCAGACTCACTCTGAAAGACTTGCCGCGGCCTTTGGACATACTTAA

Amino acid sequence of ORF of BpMYB61 from birch

MGRHSCCYKQKLRKGLWSPEEDEKLLRHITKYGHGCWSSVPKQAGLQRCGKSCRLRWINYLRPDLKRGT
FSQEEENLIIELHAVLGNRWSQIAAQLPGRTDNEIKNLWNSCLKKKLRQRGIDPVTHKPISEVENGEED
KDTETKSKDKMSVVSNELNLLKAESSRQESPIAAQGYRLEVQGNNLMTPTANNDFLHQDTSTTNCQPSE
LVGHFPLQQLNYVSNATNSNPTQWFTQSGKSFDMNSEFASMLPNSIGYKPSLTFPADNVSMSSFTVNGS
RYWEGSGAAATNSNSGSSSSAELQSNSSLLENSMFSWGLADCGTSDKETQTHLTEDIKWAEYFNNPLLM
AAALQNQTPQCLYNEIKSETHLVTDTSTAMWAHHNKQQGSLQTHSERLAAAFGHT
