# Supplementary material for: Functional identification of BpMYB21 and BpMYB61 transcription factors responding to MeJA and SA in birch triterpenoid synthesis
Source: BMC Plant Biol. 2020 Aug 12;20:374. doi: 10.1186/s12870-020-02521-1 (PMC7422618; doi:10.1186/s12870-020-02521-1)
Supplement: Supplementary file 3 — Additional file 3. Accession numbers in NCBI of all sequence data and phylogeny data in Figs. 2 and 4. [file 12870_2020_2521_MOESM3_ESM.pdf]

Genebank ID：

BpMYB21：MF574045
Populus trichocarpa：XP_002316936.1, EEE97548.1
Vitis vinifera: CAN81932.1, XP_002266049.1
Cephalotus follicularis: GAV67420.1
Eriobotrya japonica: AOQ26224.1
Prunus persica: XP_007212865.1, ONI12921.1
Populus euphratica: XP_011029391.1, XP_011014456.1
Nicotiana attenuata: XP_019229348.1, OIT30152.1
Ziziphus jujuba：XP_015896599.1
Fragaria vesca subsp. Vesca: XP_004293589.1
Nicotiana sylvestris: XP_009801248.1
Nicotiana tabacum: XP_016436111.1
Prunus mume: XP_008226723.1
Juglans regia: XP_018823636.1
Nelumbo nucifera: XP_010277911.1
Macleaya cordata: OVA07982.1
Jatropha curcas: XP_012091477.1, KDP20873.1, AIT52303.1
Hevea brasiliensis: XP_021664638.1
Manihot esculenta: XP_021606635.1, OAY54974.1

BpMYB61：KT344120
Ricinus communis: XP_015572455.1
Phaseolus vulgaris: XP_007145085.1, ESW17079.1
Glycine max: XP_003519722.1, KHN00250.1, KRH69136.1
Betula luminifera: ACJ38663.1
Populus nigra: BAR45573.1
Juglans regia: XP_018843144.1, XP_011040042.1
Epimedium sagittatum: AFH03054.1
Theobroma cacao: XP_007051325.1, EOX95482.1
Citrus clementina: XP_006444636.1, ESR57876.1
Gossypium raimondii: XP_012480219.1, KJB09540.1
Gossypium hirsutum: XP_016695803.1
Jatropha curcas: XP_012083029.1, KDP28358.1, AIT52238.1
Populus tomentosa: AIA66961.1
Populus trichocarpa: XP_002320929.1, EEE99244.1
Manihot esculenta: OAY48816.1
Vitis vinifera: XP_002281027.1
Citrus sinensis: XP_006492438.1, KDO86760.1
Lupinus angustifolius: OIV91042.1
Gossypium arboreum: XP_017612658.1, KHF98976.1
