# Supplementary material for: Functional identification of BpMYB21 and BpMYB61 transcription factors responding to MeJA and SA in birch triterpenoid synthesis
Source: BMC Plant Biol. 2020 Aug 12;20:374. doi: 10.1186/s12870-020-02521-1 (PMC7422618; doi:10.1186/s12870-020-02521-1)
Supplement: Supplementary file 4 — Additional file 4. Sequences of BpMYB21 and BpMYB61 promoters from birch. [file 12870_2020_2521_MOESM4_ESM.pdf]

Promoter sequences of the BpMYB21 from birch.

AGTGTAGTAGCATAGGTACGTAGTGTACTCCAGGAGCAGGGCATTGTATGGTTCTAGCAATTTCTTGGC
CACGGTGATGCTCTTATGCAAATCCTCTTCGTTGGTTTTGTCATTGTCAATCCTGAGGTGGACATTTCT
CCTGCAAATGAATCGGAACTGAGGGGCTGTATTGTGGAGTCCGGTCACTTGTAGCACATCACCAATACG
ATAACGGTATAGTCCTAAATCATTACCAAAAAACCTTATCATTTATGGTAATACAAAAAAGCCCAGCTT
TTGATGTTACACTGTCCTTGGTATAATCTTTCTTTTTAATCTCTACTCATGACTTTCATCGAATGTTAC
ACTCTCCGATGCTTATTACCTAATGTGACAATTGTAACTTCAGTTTACTCTCTTTCTGCAATTGCCACA
TCAAATAAATAAGCACTTGGGTGTAAAAGAGCTTATTACCTAATGTGACAGTTGCCACGTGATCTTTCT
ATTATAACGTTTTTTAAGCAATTGAGAATGTTAAAAAGCTTATCGTTAATGGTTGGTAATACAAAAAAG
AAAAGCTTTTGAAGTATAATCTTTTTCACTGACTTTCGTCTATTAGAGAAATGTTACATCAACTCTTTT
TGTTTTTTTACATTATTAAGAGCTTGTTACCTGATGTAAGTAACAATTGTCACGTCAGCTTTATACTAC
AGCGTTTTTTAATTCTCATATGCTTACTCTCTTTCTAGTGTAAAGACTGTAAAGAGGGAGATGAGATGA
GGAAGTGGAAGGTGGGAAGGTTGGTTGAGAAGGCAGATGAGATGAGGAAGGTTGCTCAAGTTCAAGCTG
AAGCATTTCATGTGCCTGTTGCTCTTTTCAATGACTTTTTCTTCGACTTCTTTCAGGTTTTTCCCACGC
TTCTTTTCTTCCCTTGATTTACATTTAGCGTTTGTTCCCTTTGTTTTTCTGAGCTCCCCCCATTCCTTT
TCTTTTCCCTGTTTAATTTGGATAAGCCCGTTTCCACTCAATATGTCTAGCGAGCGACGAAACTAGAAA
TAGAAATTCAACTTGTAACGGCTCTTCTGTTCAGAAATGAAATGTATTACTGGCATAATATCTACGATT
GGATTCAAAAAAGCGTAGAAGGGGCCAGGTTGCATCAAAAAATAATTCTAGAGGCCAAAATAAGAAGAA
AAAAAAGTACATGAACTATTTTTTGTAACAAATTTAAAGCTCAAAAAAATTTTTAGAGGTGGAGCCCCA
CCAGCCCCCCTTAAATCCGTCTACTATCAAAGTATTGATCACAAGTTTTGTGAAGATACA

Promoter sequences of the BpMYB61 from birch.

AGTGTAGAAGCATAGGCCAGATGGAGTACATGGACAAGCCATGTCTAAACCTAGAAGCAGGAGTAACAG
TGCTTGATCTAAAAGCTCAATGACATGGCTTGAATTGAGGCAGCCTAACAACCTTAATTTGAAAGACCT
CCCAACACCTCTATATCGTAGTCGAGGATCACAGATTCTGATGCTAATAACAACACCCAACCTCCTTAG
AGGTCACCTCTCTTTCAAGCAATAGCAGATGCTTTTGTGATTATGGCTTGTGTAGGGCTTATGGTCGTG
TTTGATTGGTTTCATATTAAATCTGCAATTCTTGATGCGTCTCTCTACTGTTACATGCATGGGTTTAAA
TATGCATATGTTGGGGTAAAACCCACCTGACAAAACTAGGCGCAAGTAACAAAGAGATGGGAATTTCCA
ATAATCATCCAATACTGGAACAGATAGCAAAACCATTCAAAACACTGAACTCGACCCAGTGGCTGAACA
TTGCCTATTGAACTGAAACTATATTCAGATCCCTCAAACAAAAGCAAATAGTAATAACAATAATATGAA
GCAAAAGAGCAAGAATTCAAGGCAGATAAACCGGTAACTGAAAAACTAAACAGAGCTTACAAGGTGCGA
AGGATTCATCTTTGCCTTCAATGAGGACTCTACTTGTTCCTTTGTCACCCCCATCTCCCTCTCTCTCTC
TCTCTCTCTCTCGCTAAACATCTTTTCTACTCCACAACTCCCTTAAAAATCCATTTCAGAGCCACCTCT
AGCCGTTGCTGACAGTTCTCCTAGGATTCAACAAAATTGCTGCTGTTTTAAGGATAGCTTCTCCCTCAT
AGCAGGCGGGTCTTGCTCTAAA
