# Supplementary material for: Functional identification of BpMYB21 and BpMYB61 transcription factors responding to MeJA and SA in birch triterpenoid synthesis
Source: BMC Plant Biol. 2020 Aug 12;20:374. doi: 10.1186/s12870-020-02521-1 (PMC7422618; doi:10.1186/s12870-020-02521-1)
Supplement: Supplementary file 5 — Additional file 5: Table S1. Specific primers used for gene cloning and sequence analysis. [file 12870_2020_2521_MOESM5_ESM.docx]

TableS1 Specific Primers of BpMYB21 and BpMYB61 (for Gene Clone and Sequence Analysis)

Genes 5’-3’

BpMYB21-full-F TCTCTCACTTTCTCTCCGTCT

BpMYB21-full-R CGCAATACCAACCATTAGAA

BpMYB61-full-F GCTCTAAAATGGGGAGGCACT

BpMYB61-full-R TTAAGTATGTCCAAAGGCCGC
