# Supplementary material for: Functional identification of BpMYB21 and BpMYB61 transcription factors responding to MeJA and SA in birch triterpenoid synthesis
Source: BMC Plant Biol. 2020 Aug 12;20:374. doi: 10.1186/s12870-020-02521-1 (PMC7422618; doi:10.1186/s12870-020-02521-1)
Supplement: Supplementary file 6 — Additional file 6: Table S2. Primers used for reverse transcription and quantitative real-time PCR (qRT-PCR) analysis. [file 12870_2020_2521_MOESM6_ESM.docx]

TableS2 The primers for reverse transcription and quantitative real-time PCR (qRT-PCR) analysis

Genes 5’-3’

BpMYB21-F CAGCGCGTACCAAATTTCAG

BpMYB21-R CTATCCCTCTCATCTTCAGTGC

BpMYB61-F GGTTGGAGTTTGTGGCATTG

BpMYB61-R AAGTGCAGGGTAACAATTTGATG
