# Supplementary material for: Functional identification of BpMYB21 and BpMYB61 transcription factors responding to MeJA and SA in birch triterpenoid synthesis
Source: BMC Plant Biol. 2020 Aug 12;20:374. doi: 10.1186/s12870-020-02521-1 (PMC7422618; doi:10.1186/s12870-020-02521-1)
Supplement: Supplementary file 7 — Additional file 7: Table S3. BpMYB21, BpMYB61 and GFP fusion primer design for subcellular localisation analysis. [file 12870_2020_2521_MOESM7_ESM.docx]

TableS3 BpMYB21 or BpMYB61 and GFP fusion primer design(Subcellular Localization)

Genes 5’-3’

BpMYB21-GFP-F GGACTCTTGACCATGGTCACTTTCTCTCCGTCTCTATCT

BpMYB21-GFP-R GTCAGATCTACCATGG**C**CATATGCAATCCACGGGCA

BpMYB61-GFP-F GGACTCTTGACCATGGGGTCTTGCTCTAAAATGGGG

BpMYB61-GFP-R GTCAGATCTACCATGG**C**AGTATGTCCAAAGGCCGC
