# Supplementary material for: Functional identification of BpMYB21 and BpMYB61 transcription factors responding to MeJA and SA in birch triterpenoid synthesis
Source: BMC Plant Biol. 2020 Aug 12;20:374. doi: 10.1186/s12870-020-02521-1 (PMC7422618; doi:10.1186/s12870-020-02521-1)
Supplement: Supplementary file 8 — Additional file 8: Table S4. Intron primers for BpMYB21 and BpMYB61 [file 12870_2020_2521_MOESM8_ESM.docx]

TableS4 Intron primers Design of BpMYB21 and BpMYB61

Genes 5’-3’

BpMYB21-I-F ATGGGAAAATCTCCTTGTTGTGAAA

BpMYB21-I-R ATTGGTTGGGTGGACTGGAA

BpMYB61-I-F ATGGGGAGGCACTCTTGCTGTT

BpMYB61-I-R GGCTGGCAGTTGGTTGTGGA
