# Supplementary material for: Functional identification of BpMYB21 and BpMYB61 transcription factors responding to MeJA and SA in birch triterpenoid synthesis
Source: BMC Plant Biol. 2020 Aug 12;20:374. doi: 10.1186/s12870-020-02521-1 (PMC7422618; doi:10.1186/s12870-020-02521-1)
Supplement: Supplementary file 9 — Additional file 9: Table S5. Specific primers for BpMYB21 and BpMYB61 promoter cloning. [file 12870_2020_2521_MOESM9_ESM.docx]

TableS5 Specific primers Design of BpMYB21 and BpMYB61 Promoter Cloning

Genes 5’-3’

BpMYB21-SP1 CTTATTTCCCAAAACACCGTGC

BpMYB21-SP2 AAATAGTTTGTCCAGCGAAGCC

BpMYB21-SP3 TAGGAAGGATTCGCCATCTACCGT

BpMYB61-SP1 TGAGATTCTCTTCCTCCTGCGA

BpMYB61-SP2 AATCTGCAGCTCTTCCCACACC

BpMYB61-SP3 GCTCCAACAGCCATGACCATAC
