# Supplementary material for: Functional identification of BpMYB21 and BpMYB61 transcription factors responding to MeJA and SA in birch triterpenoid synthesis
Source: BMC Plant Biol. 2020 Aug 12;20:374. doi: 10.1186/s12870-020-02521-1 (PMC7422618; doi:10.1186/s12870-020-02521-1)
Supplement: Supplementary file 10 — Additional file 10: Table S6. Primers for promoter activity analysis [file 12870_2020_2521_MOESM10_ESM.docx]

TableS6 Design of primers for promoter activity analysis of BpMYB21 and BpMYB61

Genes 5’-3’

BpMYB21-P-F CTAGTGGATCCCCAATACTAGTGTAGTAGCATAGGTACGTAGTGTAC

BpMYB21-P-R TAACATGGATCCCCAATACTTGTATCTTCACAAAACTTGTGATCAA

BpMYB61-P-F ACTAGTGGATCCCCAATACTAGTGTAGAAGCATAGGCCAGATGG

BpMYB61-P-R TAACATGGATCCCCAATACTTTTAGAGCAAGACCCGCCTG
