# Supplementary material for: Functional identification of BpMYB21 and BpMYB61 transcription factors responding to MeJA and SA in birch triterpenoid synthesis
Source: BMC Plant Biol. 2020 Aug 12;20:374. doi: 10.1186/s12870-020-02521-1 (PMC7422618; doi:10.1186/s12870-020-02521-1)
Supplement: Supplementary file 11 — Additional file 11: Table S7. Primers for construction of yeast expression vectors for squalene and total triterpenoid analysis. [file 12870_2020_2521_MOESM11_ESM.docx]

TableS7 The primers for constructs of yeast expression vector for analysis of Squalene and total triterpenoid contents

Genes 5’-3’

BpMYB21-Y-F **CAGTGTGCTGGAATTCATGGGAAAATCTCCTTGTTGTG**

BpMYB21-Y-R **GATATCTGCAGAATTCTTACATATGCAATCCACGGG**

BpMYB61-Y-F **CAGTGTGCTGGAATTCATGGGGAGGCACTCTTGCTGTT**

BpMYB61-Y-R **GATATCTGCAGAATTCTTAAGTATGTCCAAAGGCCGC**
