# Supplementary material for: Functional identification of BpMYB21 and BpMYB61 transcription factors responding to MeJA and SA in birch triterpenoid synthesis
Source: BMC Plant Biol. 2020 Aug 12;20:374. doi: 10.1186/s12870-020-02521-1 (PMC7422618; doi:10.1186/s12870-020-02521-1)
Supplement: Supplementary file 12 — Additional file 12: Table S8. Primers for component synthesis and construction of the bait vector for yeast one-hybrid assays. [file 12870_2020_2521_MOESM12_ESM.docx]

TableS8 The primers for component synthesis and constructs of Bait vector(Yeast One-Hybrid

Assay)

Genes 5’-3’

1-F ACTATAGGGCGAATTCCCGGAATTCCGG CAACTG CAACTG CAACTG CG
1-R AGCTCCCCGG GAATTCCGAGCTCG CAGTTG CAGTTG CAGTTG CCG
2-F ACTATAGGGCGAATTCCCGGAATTCCGG CGGTCA CGGTCA CGGTCA CG
2-R AGCTCCCCGG GAATTCCGAGCTCG TGACCG TGACCG TGACCG CCG
3-F ACTATAGGGCGAATTCCCGGAATTCCGG TAACTG TAACTG TAACTG CG

3-R AGCTCCCCGG GAATTCCGAGCTCG CAGTTA CAGTTA CAGTTA CCG

4-F ACTATAGGGCGAATTCCCGGAATTCCGG AACCTAAAACCTAA AACCTAA CG

4-R AGCTCCCCGG GAATTCCGAGCTCG TTAGGTT TTAGGTT TTAGGTT CCG

5-F ACTATAGGGCGAATTCCCGGAATTCCGGaaaAaaCSGTTAaaaAaaCSGTTA

aaaAaaCSGTTACG

5-R AGCTCCCCGGGAATTCCGAGCTCGTAACSGttTtttTAACSGttTttt

TAACSGttTtttCCG

6-F ACTATAGGGCGAATTCCAACGG CAACGG CAACGG CAACGG CAACGG

6-R AGCTCCCCGG GAATTCCCGTTGCCGTTGCCGTTGCCGTTGCCGTTG
