# Supplementary material for: Functional identification of BpMYB21 and BpMYB61 transcription factors responding to MeJA and SA in birch triterpenoid synthesis
Source: BMC Plant Biol. 2020 Aug 12;20:374. doi: 10.1186/s12870-020-02521-1 (PMC7422618; doi:10.1186/s12870-020-02521-1)
Supplement: Supplementary file 13 — Additional file 13: Table S9. Primers for construction of the prey vector for yeast one-hybrid assays. [file 12870_2020_2521_MOESM13_ESM.docx]

TableS9 The primers for constructs of Prey vector(Yeast One-Hybrid Assay)

Genes 5’-3’

BpMYB21-p-F TGGCCATTATGGCCCGGG ATGGGAAAATCTCCTTGTTGTG

BpMYB21-p-R GACATGTTTTTTCCCGGG TTACATATGCAATCCACGGG

BpMYB61-p-F TGGCCATTATGGCCCGGG ATGGGGAGGCACTCTTGCTGTT

BpMYB61-p-R GACATGTTTTTTCCCGGG TTAAGTATGTCCAAAGGCCGC
